# First Characterization of a *Hafnia* Phage Reveals Extraordinarily Large Burst Size and Unusual Plaque Polymorphism

**DOI:** 10.3389/fmicb.2021.754331

**Published:** 2022-02-08

**Authors:** Lingting Pan, Dengfeng Li, Zhitong Sun, Wei Lin, Binxin Hong, Weinan Qin, Lihua Xu, Wencai Liu, Qin Zhou, Fei Wang, Ruqian Cai, Minhua Qian, Yigang Tong

**Affiliations:** ^1^Key Laboratory of Marine Biotechnology, School of Marine Sciences, Ningbo University, Ningbo, China; ^2^College of Life Science and Technology, Beijing University of Chemical Technology, Beijing, China

**Keywords:** *Hafnia* phage, genome, big burst size, polymorphism, HNH homing endonuclease

## Abstract

A unique lytic phage infecting *Hafnia paralvei* was isolated and identified. *Hafnia* phage Ca belongs to the family Autographiviridae, possessing an icosahedral head with a diameter of 55 nm and a short non-contractile tail. Unusually, the burst size of *Hafnia* phage Ca of 10,292 ± 1,097 plaque-forming units (PFUs)/cell is much larger than other dsDNA phages reported before. Compared to the genome of the related phage, *Hafnia* phage Ca genome contains extra genes including DNA mimic ocr, dGTP triphosphohydrolase inhibitor, endonuclease, endonuclease VII, and HNH homing endonuclease gene. Extraordinarily, the phage developed different sizes of plaques when a single plaque was picked out and inoculated on a double-layer Luria broth agar plate with its host. Furthermore, varied packaging tightness for the tails of *Hafnia* phage Ca was observed (tail length: 4.35–45.92 nm). Most of the tails appeared to be like a cone with appendages, some were dot-like, bun-like, table tennis racket handle-like, and ponytail-like. Although the complete genome of *Hafnia* phage Ca is 40,286 bp, an incomplete genome with a deletion of a 397-bp fragment, containing one ORF predicted as HNH homing endonuclease gene (HEG), was also found by high throughput sequencing. Most of the genome of the virus particles in large plaques is complete (>98%), while most of the genome of the virus particles in small plaques is incomplete (>98%), and the abundance of both of them in medium-sized plaques is similar (complete, 40%; incomplete, 60%). In an experiment to see if the phage could be protective to brocade carps intramuscularly injected with *H. paralvei* LY-23 and phage Ca, the protection rate of *Hafnia* phage Ca to brocade carp (*Cyprinus aka* Koi) against *H. paralvei* was 33.38% (0.01 < *p* < 0.05). This study highlights some new insights into the peculiar biological and genomic characteristics of phage.

## Introduction

*Hafnia* are widespread Gram-negative facultatively anaerobic bacteria. *Hafnia* includes two known species (*Hafnia alvei* and *Hafnia paralvei*) belonging to the Enterobacteriaceae family (Janda and Abbott, 2006; Huys et al., 2010). It is normally isolated from the gastrointestinal tract of animals and humans and from food (Osuka et al., 2011). There are a number of reports on diseases associated with *H. alvei* and *H. paralvei* involved in a variety of animal and human infections (Real et al., 1997; Casagrande Proietti et al., 2004; Casanova-Román et al., 2004; Janda and Abbott, 2006; Osuka et al., 2011; Padilla et al., 2013; Orozova et al., 2014; Jayol et al., 2017; Litrenta and Oetgen, 2017; Rodríguez-Alarcón et al., 2019; Yin et al., 2019; Zhang et al., 2019; Cutuli et al., 2021). The main symptoms of *H. alvei* diseases included hepatosplenomegaly, multifocal nectrotizing hepatitis, and splenitis (Real et al., 1997; Casagrande Proietti et al., 2004; Orozova et al., 2014). In the aquaculture industry, *H. alvei* was found to be the cause of epizootic outbreaks of septicemia in rainbow trout (Gelev et al., 1990), brown trout (Orozova et al., 2014), and bacterial kidney disease of cherry salmon (Teshima et al., 1992). An *H. paralvei* strain LY-23 (MCCC 1K06097) was found to be associated with diseased turbot fish in Liaoning Province in 2015 and isolated by Professor Fengling Bai of Bohai University. In this study, *H. paralvei* LY-23 was found to be multidrug-resistant (MDR) and to cause mortality in brocade carp (*Cyprinus aka* Koi) for the first time. Alternatives of antibiotics are needed to treat infections by the MDR strain.

Bacteriophages have been considered as potential antibiotic substitutes to treat infectious diseases (Fathima and Archer, 2021). Bacteriophages are viruses that infect bacteria. They are widely distributed in nature (Jurczak-Kurek et al., 2016). With the increasingly serious and global spread of antimicrobial resistance, lytic phages are considered one of the most promising alternatives to antibiotics as they can kill pathogenic bacteria without harming their human or animal hosts (Düzgüneş et al., 2021). In recent years, more and more reports have proved the effectiveness of phage therapy in the treatment of bacterial infections for human and aquatic animals (Veyrand-Quirós et al., 2020; Donati et al., 2021; Xu et al., 2021). Only six *Hafnia* phage genomes were found in database searching, including five phages belonging to the Myoviridae family and one phage belonging to the Drexlerviridae family. Five of them are phages infecting *H. paralvei* and the other one infecting *Hafnia* spp. Genome sequence information alone of the six *Hafnia* phages can be found, but no information about plaque morphology, phage morphology, host range, stability, one-step growth curve, and the therapeutic application of *Hafnia* phage has been reported in the current literature. In other words, there just are not any *Hafnia* phages that are sufficiently characterized for phage therapy. In this study, a virulent phage, *Hafnia* phage Ca, was isolated with *H. paralvei* LY-23 as host from Guanshan River in Ningbo, Zhejiang Province, China. *Hafnia* phage Ca revealed an apparent protective effect on brocade carps against *H. paralvei* infection.

The therapeutic value of a phage depends on the characterization of phage properties such as stability, growth kinetics, host range, and viral yield (Jacquemot et al., 2018). Usually, a phage stock contains a uniform genome and morphology and forms uniform plaques. Most reported phages are exclusively host strain-specific. A minority of phages infect bacteria at the species level. A few phages infect bacteria at the genus level, and a few phages infect bacteria of different genera, such as phage vB_EcoM_APEC (Deng et al., 2021). The burst size of most of the reported phages, including phages of family Autographiviridae, was dozens to hundreds of PFU/cell (Baudoux et al., 2012; Huang et al., 2013; Merabishvili et al., 2014; Qin et al., 2017; Anand et al., 2018; Lu et al., 2020). A literature review found that the biggest burst size of the reported dsDNA phages is *Cronobacter* phage vB_CtuP_B1 of the Autographiviridae family, which has a particularly high burst size of 4,006 PFU/cell (Zeng et al., 2021). Here we characterize the *Hafnia* phage Ca, describing its polymorphism and large burst size.

## Materials and Methods

### Bacterial Strain

*Hafnia paralvei* LY-23 was kindly provided by Professor Fengling Bai of Bohai University. It was deposited in the Marine Culture Collection of China (MCCC) with accession no. MCCC 1K06097. *H. paralvei* LY-23 was grown in LB medium (5 g/L yeast extract, 10 g/L tryptone, 5 g/L NaCl) at 29°C with a shaking speed of 220 rpm.

### Antibiotic Susceptibility Test

An antibiotic susceptibility test of *H. paralvei* LY-23 was conducted using antibiological susceptibility disks (Hangzhou Binhe Microorganism Reagent. Co., Ltd., Hangzhou, China), according to the instructions and following the standard of the Clinical and Laboratory Standards Institute (CLSI). Seventeen types of antibiotic disks [penicillin G (6 μg), amoxicillin (10 μg), cephalexin (30 μg), kanamycin (30 μg), gentamicin (10 μg), tobramycin (10 μg), azithromycin (15 μg), aboren (30 μg), chloramphenicol (30 μg), ofloxacin (5 μg), tetracycline (30 μg), doxycycline (30 μg), rifampin (5 μg), trimethoprim/sulphamethaxazole (1.25/23.75 μg), vancomycin (30 μg), polymyxin (30 μg), and clindamycin (2 μg)] were used. An *H. paralvei* LY-23 culture in the exponential growth phase (2.56 × 10^7^) was spread by sterile swab on Muller–Hinton agar plates. After drying at room temperature, antibiotic disks were then placed on the surface of the medium in triplicates. The plates were then incubated at 29°C for 16–18 h. The diameters of the formed inhibition zones were measured.

### Phage Isolation

A surface water sample was collected from Guanshan River (North latitude, 29.966204; East longitude, 121.452744) in Cicheng Town of Ningbo City in Zhejiang Province, China. The collected sample was centrifuged at 10,000 × *g* for 10 min at 4°C; 40 ml of the supernatant, 20 ml of 3 × LB medium, and 1 ml of *H. paralvei* LY-23 culture in the exponential growth phase (2.56 × 10^7^ CFU/ml) were mixed and incubated at 29°C with shaking speed of 180 rpm for 3 h. The culture was centrifuged at 10,000 × *g* for 10 min at 4°C, and the supernatant was filtered sequentially through 0.45 μm and 0.22 μm nitrocellulose microporous filters (ANPEL Laboratory Technologies Inc., Shanghai, China); 4 ml of filtrate and 2 ml of 3 × LB medium were mixed with 100 μl of *H. paralvei* LY-23 in the exponential growth phase (2.56 × 10^7^ CFU/ml), and cocultured at 29°C with shaking speed of 180 rpm until the obvious phenomenon of bacteriolysis was observed. The lysate was centrifuged at 10,000 × *g* for 10 min at 4°C, and the supernatant was taken to purify phage strain via five-serial single-plaque isolation using the conventional double-layer agar method (Zhang et al., 2017). Briefly, phage suspension was serially diluted (10^–1^–10^–9^) with LB; 100 μl of each dilution was mixed with 200 μl *H. paralvei* LY-23 of log phase (OD600≈0.6, 2.56 × 10^7^ CFU/ml), and allowed to adsorb for 10 min at 29°C. The mixture was then added to 3 ml of melted 0.7% agar LB medium (preheated at 45°C), mixed quickly, and poured onto a 1.5% agar LB plate. A single large plaque, a single medium-sized plaque, and a single small plaque were picked from the plate with appropriate plaque density, respectively; suspended in 5 ml of the logarithmic phase *H. paralvei* LY-23; and cultured at 29°C with shaking speed of 180 rpm until obvious lysis. The lysate was centrifuged at 10,000 × *g* for 10 min at 4°C, and the supernatant was filtered sequentially through 0.45 μm and 0.22 μm nitrocellulose microporous filters. The filtrate was used for a new round of plaque isolation. Successive single-plaque isolation was carried out eight times using large, medium, and small plaques. Plaques on each plate of each generation were always of different sizes. The *Hafnia* phage Ca, picked from a purified single-large plaque of the fifth generation, was deposited in the China General Microbiological Culture Collection Center under number CGMCC No. 17097 and used for morphology observation, proliferation kinetic, and stability analysis, genome sequencing.

In addition, 14 times the amount of successive single-large plaque was also picked, and the virions in the 14th large plaque, the 14th medium plaque, and the 14th small plaque were used to develop plaques on a double-layer plate with an *H. paralvei* LY-23 lawn. The 15th large-, medium-, and small-sized plaques on each Luria broth agar *H. paralvei* LY-23 lawn were counted. The ratio of different-size plaques originated from virions in the 14th large-, medium-, and small-size plaques were calculated respectively. This experiment was carried out in triplicates.

### Transmission Electron Microscopy

For electron microscopy observation, *Hafnia* phage Ca suspensions were centrifuged at 12,000 × *g* for 15 min at 4°C, and the supernatant was further centrifuged at 58,000 × *g* for 1 h at 4°C. The sediment was washed twice with phosphate-buffered saline (PBS) and resuspended in PBS, deposited on carbon-coated copper grids for 10 min, stained with 3% uranyl acetate for 20 s, and observed under a transmission electron microscopic (Hitachi-7650; Hitachi, Tokyo, Japan).

### Host Range Test

The host range of *Hafnia* phage Ca was tested against 37 bacterial strains (Table 1) using the spot test as described (Ghosh et al., 2018). In total, 200 μl of tested bacterial cells was added to each of the 3 ml of melted 0.7% agar medium (Pre-incubated at 45°C), mixed quickly, and poured onto 1.5% agar medium to make double-layered agar plates. After solidification, 5 μl of phage suspension (2.27 × 10^6^ PFU/ml) was spotted onto the double-layer plates in triplicates. The plates were incubated at 29°C overnight. Clear zones at the spot areas indicated positive susceptibility. To further verify the results, the host range of *Hafnia* phage Ca was also tested against the 37 bacterial strains using 96-well cell culture plates. In total, 200 μl of the logarithmic phase bacteria (OD600≈0.6) was added to each well, and 20 μl phage suspension (6.93 × 10^9^ PFU/ml) was added as test group in triplicates, or 20 μl LB medium was added as control group. The 96-well cell culture plates were incubated at 29°C overnight with a shaking speed of 180 rpm. The titer (plaque forming unit/ml, PFU/ml) of each well was measured using the double-layer agar method (Stephenson, 2010).

**TABLE 1:** The results of the host range analysis test of *Hafnia* phage Ca.

Strain	Infectivity
*Hafnia alvei* LY-23	+
*Aeromonas sobria* ATCC43979	+
*Hafnia psychrotolerans* CGMCC 1.12806	−
*Hafnia alvei* CGMCC 1.2026	−
*Aeromonas hydrophila* ATCC49140	−
*Aeromonas hydrophila* A18	−
*Aeromonas hydrophila* AV4	−
*Aeromonas hydrophila* AS1 18031	−
*Aeromonas hydrophila* Ah2	−
*Aeromonas salmonicida* CGMCC 1.16015	−
*Aeromonas veronii* CGMCC 1.927	−
*Aeromonas Veyron*	−
*Vibrio harveyis*	−
*Vibrio harveyi* 1-5	−
*Vibrio alginolyticuss*	−
*Vibrio alginolyticus* WY	−
*Edwardsiella tarda* ET	−
*Shewanella putrefaciens*	−
*Staphylococcus aureus*sa 69	−
*Proteus vulgaris*	−
*Proteus mirabilis*	−
*Enterobacter sakazakii*	−
*Bacillus cereus*	−
*Shigella sonnei*	−
*Shigella dysenteriae*	−
*Enterobacter cloacae*	−
*Listeria monocytogenes*	−
*Salmonella typhosa*	−
*Salmonella paratyphi*	−
*Escherichia coli* DH5α	−
*Escherichia coli*	−
*Pseudomonas aeruginosa*	−
*Citrobacter freundii*	−
*Pseudomonas putida*	−
*Lactococcus garvieae*	−
*Vibrio Parahemolyticus* MCCC 1A11655	−
*Vibrio anguillarum*	−

*+, Susceptible; −, not susceptible.*

Additionally, the titers of a phage suspension (6.2 × 10^9^ PFU/ml) on susceptible bacteria strains were tested in triplicates using the double-layer agar method. According to Guo et al. (2021), EOP, the ratio of the phage titer on the test strain to the phage titer obtained from the reference bacteria strain (*H. paralvei* LY-23) was calculated and recorded as the mean ± standard deviation (SD).

### Optimal Multiplicity of Infection, Optimum Adsorption Time, and One-Step Growth Curve

The optimal multiplicity of infection (MOI) was tested according to the reference (Wang et al., 2014); 2.56 × 10^7^ CFU of LY-23 in logarithmic phase was mixed with a set of serial dilutions of *Hafnia* phage Ca suspension at MOIs of 0.0001, 0.001, 0.01, 0.1, 1, and 5, respectively, in triplicates. After 10 min of absorption at 29°C, the mixtures were centrifuged at 10,000 × *g* for 10 min. The sediments were washed twice with LB, resuspended in an equal volume of LB medium, and incubated at 29°C for 4 h with a shaking speed of 180 rpm. Samples were collected and centrifuged at 10,000 × *g* for 10 min. Titers in the supernatant of the samples were measured by using the double-layer agar plate method (Gallet et al., 2009). The experiment was repeated three times. The MOI with the highest phage production was considered as the optimal one.

To test the optimum adsorption time, the phage Ca was mixed with LY-23 (4.7 × 10^8^ CFU/ml) at the optimal MOI of 0.001 in triplicate and incubated at 29°C with a shaking speed of 180 rpm. Samples were collected at 0, 2, 4, 6, 8, 10, 15, and 20 min post-inoculation and centrifuged at 10,000 × *g* for 10 min at 4°C. Phage titers in the supernatant of the samples were measured by using the double-layer agar plate method. The adsorption rate was calculated by the ratio of the difference between average phage titers at 0 min and average phage titers at sampling time to average phage titers at 0 min.

The latent period and burst size of phage Ca was determined by a one-step growth curve. Each 400 μl of phage suspension (4.7 × 10^7^, 4.7 × 10^10^, 2.4 × 10^11^ PFU/ml) was mixed with 40 ml *H. paralvei* strain LY-23 suspension (4.7 × 10^8^ CFU/ml) at the optimal MOI of 0.001, 1, and 5, respectively, in triplicates. The mixtures were incubated at 29°C for 2 min to allow phage adsorption and centrifuged at 10,000 × *g* for 10 min. The sediments were washed twice with LB, suspended in an equal volume of LB, and then cultured at 29°C with a shaking speed of 220 rpm. Samples were collected at 0, 10, 20, 30, 40, 50, 60, 90, 120, 150, 180, 210, and 240 min. Phage titers in the samples were determined using a double-layer agar method with three repetitions.

Two other methods were used to quantify the phages in the samples collected from the co-cultures under the optimal MOI of 0.001. One method to determine the phage numbers was SYBR Green I-based quantitative PCR (qPCR). The forward primer and reverse primer (5′-GGCAAGAAGAAATGACCCAGAG-3′ and 5′-ATCTGACATCTGGTTGGGACTG-3′) targeted to the terminase large subunit (TerL) gene were designed and used. The positive standard phage DNA was extracted from a sample collected at 240 min, using the Hipure Lambda Mini Kit (Magen, product no. P1161-02). The concentration of phage DNA standard was detected using NanoDrop 2000 (Thermo Fisher Scientific Inc., Wilmington, DE, United States). The phage copy concentration was equal to the DNA concentration divided by the molecular weight of the phage genome; 500 μl of each sample collected at the 13 time points were treated with DNase I (final concentration, 1 μg/ml) at 37°C for 2 h, incubated at 80°C for 15 min, and were then applied to extract phage DNA with the Hipure Lambda Mini Kit (Magen, product no. P1161-02). Real-time PCRs were performed to detect the 10-fold dilution series of positive standard phage DNA and the samples at each time point. A standard curve was established based on the detection results of the dilution series of positive standard phage DNA. The phage copy concentrations in the samples at the 13 time points were calculated based on the standard curve and the qPCR results in triplicates. One-step growth curves were drawn using GraphPad Prism 7. Finally, the burst size of the *Hafnia* phage Ca was calculated as the ratio of the number of released virions to the number of infected bacterial cells in the initial latent period according to the reference (Hyman and Abedon, 2009; Casey et al., 2015; Xu et al., 2018; Imam et al., 2019). The second method (Casey et al., 2015) was dedicated to the calculation of free phages. Samples were centrifuged (10,000 × *g* for 10 min). Supernatants were filtered through 0.45 μm and 0.22 μm filters successively. Phage titers in the filtrates were determined using the double-layer agar method with three repetitions.

The latent period and burst size of phage Ca against *Aeromonas sobria* ATCC43979 was also determined by a one-step growth curve. In the initial step of the experiment to develop a one-step growth curve, the 40 ml *H. paralvei* strain LY-23 suspension (4.7 × 10^8^ CFU/ml) was replaced with isochoric *A. sobria* ATCC43979 suspension (1.17 × 10^7^ CFU/ml).

### Temperature, pH, and Chloroform Sensitivity

For temperature sensitivity assessment, a series of *Hafnia* phage Ca suspensions (1 × 10^10^ PFU/ml) were incubated at 40, 60, and 80°C, respectively, in triplicates, and the phage titers at 30, 60, 90, and 120 min were analyzed using the double-layer agar method. For pH stability test, a series of *Hafnia* phage Ca suspensions (1 × 10^10^ PFU/ml) were adjusted to pH 2, 3, 4, 5, 6, 7, 8, 9, 10, 11, and 12 using NaOH or HCl in triplicates, incubated at 29°C for 2 h, and the phage titers were analyzed using the double-layer agar method. For testing the sensitivity of the *Hafnia* phage Ca chloroform, the phage suspensions (1.86 × 10^11^ PFU/ml) were added chloroform to 2.5% in triplicates and incubated at 29°C for 2 h. Isovolumetric PBS (0.01 M) was added instead of chloroform in control groups. Phage titers were analyzed by the double-layer agar method. The experiment was repeated three times.

### Genome Sequencing and Analysis

Phage cultures from large-, medium-, and small-size plaques were separately sequenced. The phage *Hafnia* phage Ca suspension was centrifuged for 20 min at 10000 × *g*, filtered through a 0.22 μm nitrocellulose filter, pretreated with DNase and RNase; 400 μl of filtrate was used for phage genome extraction with the High Pure Viral kit (Roche, Product No.: 11858882001). The kit allows the extraction of DNA and RNA together. To clarify the type and composition of the nucleic acid extracted from the phage suspension, the nucleic acid was treated with DNase I, RNase A, and S1 nuclease at 37°C for 30 min, respectively, and detected by 1% agarose gel electrophoresis. A DNA library was constructed with the NEBNext Ultra II DNA Library Prep Kit for Illumina. Sequencing of the phage was performed on an Illumina MiSeq (San Diego, CA, United States) sequencer to obtain paired-end reads. Then, Trimmomatic-0.36 was used for filtering out the low-quality (*Q*-value < 20) reads and adapters. At last, assembling was performed using Newbler v3.0 software. The genomic termini were predicted according to the described method (Zhang et al., 2015). The promoter was predicted using the Softberry server^1^. ORFs were annotated by the RAST website server^2^ (Aziz et al., 2008) and identified with HMMER^3^ (Potter et al., 2018) (*E*-value ≤ 10^––5^) and HHpred server^4^ (Zimmermann et al., 2018) (*E*-value ≤ 10^–5^, percentage possibility of homologous sequences > 96%). tRNA was predicted by using tRNAscan-SE software^5^ (Schattner et al., 2005) in the *Hafnia* phage Ca genome.

### Taxonomic Analysis

Preliminary nucleotide sequence comparisons were made using BLASTn server of NCBI. The average nucleotide identity (ANI) value between the *Hafnia* phage Ca and the most related phage ST31 (NC_047829.1) was calculated using the EzGenome web server^6^ (Figueras et al., 2014). DNA-DNA hybridization (DDH) is a technique based on comparative analysis between the total DNA of two species. This genome-based method is used as the taxonomic standard for species delineation. If the DDH value of the genomic DNA of two respective organisms is below 70%, they are regarded as distinct species (Figueras et al., 2014). The *in silico* DNA-DNA hybridization (isDDH) identity between the *Hafnia* phage Ca and *Escherichia* phage ST31 was calculated using the GGDC web server^7^ (Meier-Kolthoff et al., 2013). The Pairwise Sequence Comparison (PASC) tool^8^ (Bao et al., 2014) was used to calculate nucleotide sequence similarity between *Hafnia* phage Ca and other phages in current databases (Jul 13, 2021). ViPTree online (Nishimura et al., 2017)^9^ was used to generate a proteomic tree based on genome-wide sequence similarities, computed by tBLASTx, for the 56 classified phages of the 23 genera of subfamily Studiervirinae of family Autographiviridae and *Hafnia* phage Ca.

### Protective Effects of the *Hafnia* Phage Ca Against *Hafnia paralvei* in Brocade Carp

A protective experiment of *Hafnia* phage Ca against *H. paralvei* in brocade carp was performed under two conditions: without aeration and continuous aeration.

The experiment under the condition without aeration was as follows: 54 healthy brocade carps with a length of 7–8 cm were randomly divided into three groups without aeration. Each fish of the blank group was successively injected intraperitoneally twice with 0.01 M PBS. Each fish of the control group was successively injected intraperitoneally with *H. paralvei* LY-23 (10^7^ CFU/ml) and 0.01 M PBS. Each fish of the test group was successively injected intraperitoneally with *H. paralvei* LY-23 (10^7^ CFU/ml) and *Hafnia* phage Ca (10^6^ PFU/ml). The injection time interval was 2 h and the injection volume was 50 μl. Death occurred in the second to the fifth day. The fish mortality of each group was recorded daily.

The experiment under the condition with continuous aeration was as follows: a total of 90 brocade carps (average length: 7 cm) were randomly divided into three groups in triplicates with continuous aeration. Each fish of the blank groups was intramuscularly injected with 100 μl of 0.01 M PBS twice with an interval time of 2 h. Each fish of the control groups was successively injected intramuscularly with 100 μl of *H. paralvei* LY-23 (10^7^ CFU/ml) and 100 μl of 0.01 M PBS with an interval time of 2 h. For phage therapy, each brocade carp of the experimental groups was injected successively with 100 μl of *H. paralvei* LY-23 (10^7^ CFU/ml) and 100 μl of *Hafnia* phage Ca (10^6^ PFU/ml) with an interval time of 2 h. Death occurred from the second to the seventh day. The cumulative mortality of each group was recorded daily for 7 days. The protection rate was calculated by the ratio of the difference between average mortality of the control groups and average mortality of the test groups to average mortality of the control groups.

All statistical analyses were performed using SPSS. Results were expressed as means ± standard deviation (SD). One-way ANOVA was used to determine the statistical significance, and the significance level was defined as *p ≤ 0.05 and ^**^p ≤ 0.01.

## Results

### Antibiotic Susceptibility of *Hafnia paralvei* LY-23

*Hafnia. paralvei* LY-23 was resistant to 7 of 17 tested antibiotics, which were cephalexin, penicillin G, amoxicillin, aboren, clindamycin, vancomycin, and rifampicin (Supplementary Figure 1). Its sensitivity was intermediate to kanamycin, azithromycin, and doxycycline. Bacterial strains resistant to three or more antimicrobials categories were defined as multidrug-resistant (MDR) (Magiorakos et al., 2012). *H. paralvei* LY-23 is a multidrug-resistant strain.

### Plaque and Phage Morphology

**FIGURE 1:**
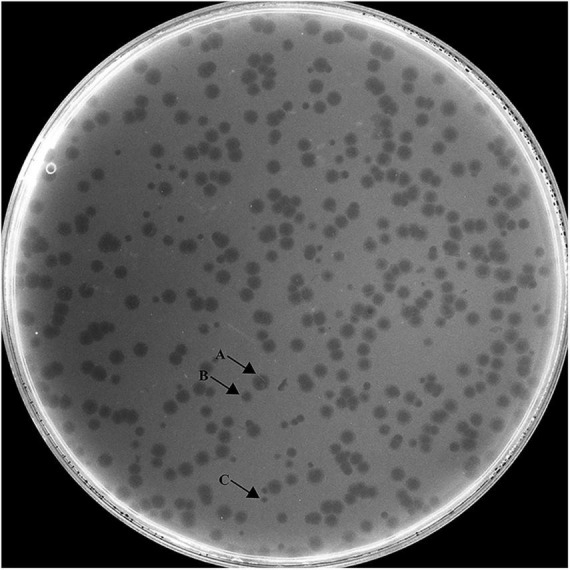
Morphology of *Hafnia* phage Ca plaques. *Hafnia* phage Ca always developed variable sizes of plaques (at least three obviously different sizes) when a single plaque was picked out and inoculated on a double-layer Luria broth agar plate with *H. paralvei* LY-23. Arrow A indicates large plaque, Arrow B indicates medium-sized plaque, and Arrow C indicates small plaque.

**FIGURE 2:**
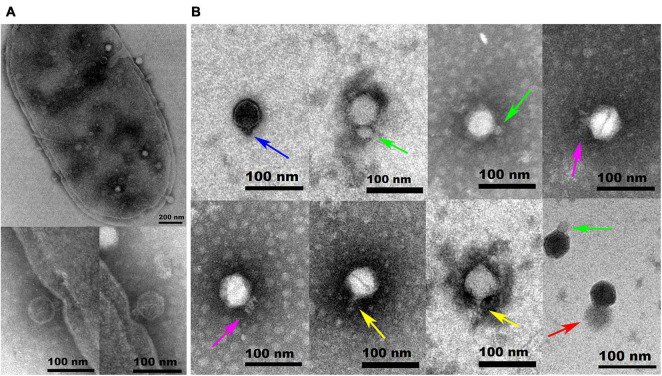
Transmission electron microscopy images of negatively stained *Hafnia paralvei* LY-23 cell infecting *Hafnia* phage Ca **(A)** and *Hafnia* phage Ca **(B)**. *Hafnia* phage Ca particles possessed an icosahedral head with a diameter of 55 nm and a short tail with a length of 4.35–45.92 nm. The tail packaging tightness of *Hafnia* phage Ca is variable, presenting dot-like (blue arrow), bun-like (green arrow), cone-like with appendages (purple arrow), table tennis racket handle-like (yellow arrow), or ponytail-like (red arrow) shape. Each bun-like tail contains a visible tip sting and may be the tightened state of cone-like tails with appendages. Negative staining and electron microscopy observation, though, were redone four times in different laboratories and consistent results were obtained. The possibility cannot be ruled out that some differences might be artifacts. Cryo-EM observation could be a useful research direction for the phage in the future. The invading phages on the cell surface and the virus particles packaged in the cell are clearly visible **(A)**. The cell wall in two places (see the enlarged photos at the bottom left for details) was invaginated due to the penetration of *Hafnia* phage Ca with the cone-like tails.

*Hafnia* phage Ca can produce plaques of different sizes (at least three obviously different sizes) when a single plaque was picked out and inoculated on a double-layer Luria broth agar plate with *H. paralvei* LY-23 (Figure 1). A similar phenomenon had only been found to be previously described on *Stenotrophomonas* phage IME13 of *Tulanevirus* genus of the Myoviridae family (Fan et al., 2012). Among the 15th generation plaques of phage Ca developed by virions from a 14th generation large plaque, the percentages of large, medium, and small plaques were 92.96% ± 5.62%, 6.34% ± 5.21%, and 0.71% ± 0.63%, respectively. Among the 15th generation plaques of phage Ca developed by virions from a 14th generation medium-sized plaque, the percentages of large, medium, and small plaques were 80.55% ± 3.58%, 12.77% ± 1.48%, and 6.68% ± 2.17%, respectively. Among the 15th generation plaques of phage Ca developed by virions from a 14th generation small plaque, the percentages of large, medium, and small plaques were 83.20% ± 2.80%, 14.96% ± 2.88%, and 1.84% ± 0.52%, respectively. Transmission electron microscopy showed that the intact *Hafnia* phage Ca particles possessed an icosahedral head with a diameter of 52.98 ± 5.20 nm and a short tail with a length of 4.35–45.92 nm (Figure 2). This is similar to the Autographiviridae phage possessing a small (ca. 60 nm in diameter) icosahedral head attached to a short tail. Although the tails of Autographiviridae phages were diverse in literature, *Hafnia* phage Ca is unique. Extraordinarily, the tail packaging tightness of *Hafnia* phage Ca is variable (Figure 2B). Most of the tails revealed a cone with appendages (purple arrow) (19.15–23.41 nm in length, 28.90–31.00 nm in width); some were dot-like (blue arrow) (4.35 nm in length, 9.04 nm in width), bun-like (green arrow) (18.74–23.41 nm in length, 16.86–26.05 nm in width), table tennis racket handle-like (yellow arrow) (26.58–30.23 nm in length, 9.92–10.81 nm in width), and ponytail-like (red arrow) (about 45.92 nm in length, 49.39 nm in width). Each bun-like tail contains a visible tip sting and may be the tightened state of cone-like tails with appendages. Although, negative staining and electron microscopy observation were redone four times in different laboratories and consistent results were obtained. The possibility cannot be ruled out that some differences might be artifacts. Cryo-EM observation could be a useful research direction for the phage in the future.

### Host Range of *Hafnia* Phage Ca

The host range of *Hafnia* phage Ca was tested against the 37 bacterial strains using both 96-well cell culture plate and spot test. The results obtained by the two methods are consistent. Results showed that *Hafnia* phage Ca could only lyse *H. paralvei* LY-23 and *Aeromonas sobria* ATCC43979 among all tested strains (Table 1). The EOP value of *Hafnia* phage Ca was 0.82 ± 0.11, indicating higher infection efficiency of *Hafnia* phage Ca against *H. paralvei* LY-23 than *A. sobria* ATCC43979. One-step growth curve demonstrated that the latent period of *Hafnia* phage Ca against *A. sobria* ATCC43979 was 2–12 min. The burst size of phage Ca against *A. sobria* ATCC43979 is the ratio of the released number of released virions (3.58 × 10^10^ ± 2.60 × 10^9^) to the number of infected *A. sobria* ATCC43979 (3.05 × 10^6^ ± 1.5 × 10^5^), which is 11,766 ± 1,032 PFU/cell (Supplementary Figure 2).

### Optimal MOI Selection, Optimum Adsorption Time, and One-Step Growth Curve

**FIGURE 3:**
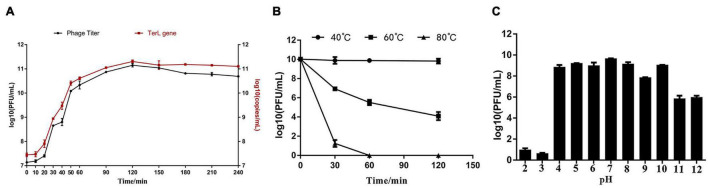
**(A)** The one-step growth curve developed under the MOI of 0.001 showed that the latent period of *Hafnia* phage Ca was between 2 and 12 min, and its burst size was 10,292 ± 1,097 PFU/cell. **(B)** The influence of temperature on *Hafnia* phage Ca activity. The activity of phage Ca is stable at 40°C. The titer of phage Ca decreased continuously at 60°C (*p* < 0.01). At 80°C, the titer dropped sharply to below the detection limit in 60 min (*p* < 0.01). **(C)** The effect of pH on the activity of *Hafnia* phage Ca. *Hafnia* phage Ca was found to be stable at pH 4–10, relatively stable at pH 11–12, and unstable at pH 2–3.

The phage titers after proliferation under MOIs of 0.0001, 0.001, 0.01, 0.1, 1, and 5 were about 5.4 × 10^10^, 1.7 × 10^11^, 6.1 × 10^10^, 4.6 × 10^10^, 1.2 × 10^11^, and 1.3 × 10^11^ PFU/ml, respectively (Supplementary Figure 3). Among all the tested MOIs, the optimal MOIs with the higher phage production were 0.001, 1, and 5. Although the phage yields under the three MOIs were approximate, based on the principle of economy, MOI of 0.001 was considered as the optimal one. Growth curve experiments were performed under the optimal MOI of 0.001. As shown in Figure 3A, the trend of the two one-step growth curves, established based on phage titers and TerL copies, respectively, is basically consistent. Adsorption of Ca to *H. paralvei* LY-23 is very efficient. The adsorption rate can reach 97.60% ± 0.23% in 2 min at 29°C (Supplementary Figure 4). The one-step growth curves demonstrated that the latent period of *Hafnia* phage Ca against *H. paralvei* LY-23 was 2–12 min, followed by a burst period of 110 min (Figure 3A). The burst size of phage Ca at MOI of 0.001 was calculated as the ratio of the final count of liberated virions at the end of the burst period (1.42 × 10^11^ ± 1.5 × 10^10^) to the initial count of infected bacterial cells at the beginning of the latent period (1.38 × 10^7^ ± 2.00 × 10^5^), which is 10,292 ± 1,097 PFU/cell. Such a big burst size of dsDNA phage had not yet been reported. The proliferation of phage Ca in *H. paralvei* LY-23 was indeed very efficient. The phage titers of phage Ca at the plateau phase were over 10^11^, while that of most dsDNA phages were reported to be 10^5^–10^10^. Quantitative fluorescent PCR detection on phage DNA standards resulted in the standard curve formula: *y* = −2.346*x* + 28.46 (*R*^2^ > 0.99, the closer the *R* squared value gets to 1, the more reliable the standard curve) (Supplementary Figure 5), which was established based on the linear relationship between log (initial copy number of template DNA) and threshold cycle value. The result was similar to the result of the double-layer agar method (Figure 3A), with the burst size of 14,708 ± 1534 copies/cell, which was calculated as the ratio of the copies’ number of released virions (2.03 × 10^11^ ± 2.10 × 10^10^) to the number of infected bacterial cells (1.38 × 10^7^ ± 2.00 × 10^5^) in the initial latent period.

As unusually high burst sizes were obtained, to rule out secondary infections artificially inflating burst size numbers, higher MOI (1 and 5) curves were developed. The proliferation of phage Ca in *H. paralvei* LY-23 under MOI 1 and 5 was also very efficient. The phage titers of phage Ca at the plateau phase were also over 10^11^. The burst size of phage Ca at MOI of 1 and 5 were 8,605 ± 245 and 3,713 ± 130 PFU/cell, respectively (Supplementary Figures 6, 7). The latent and rise periods agreed across the experiments under MOI of 0.001, 1, and 5. In addition, the titer curve of free phage virions confirmed the proliferation kinetics and unusually high yield of *Hafnia* phage Ca (Supplementary Figure 8).

### Temperature Stability, pH Stability, and Chloroform Sensitivity

The activity of *Hafnia* phage Ca is stable at 40°C (Figure 3B). The titer of *Hafnia* phage Ca decreased continuously at 60°C (*p* < 0.01). At 80°C, the titer was below the detection limit in 60 min (*p* < 0.01). As shown in Figure 3C, *Hafnia* phage Ca was found to be stable at pH 4–10, relatively stable at pH 11–12, unstable at pH 2–3. *Hafnia* phage Ca is resistant to chloroform, as the average phage titer of the chloroform treat groups was 1.86 × 10^11^ PFU/cell, consistent with the untreated control group.

### Genome Analysis

**FIGURE 4:**
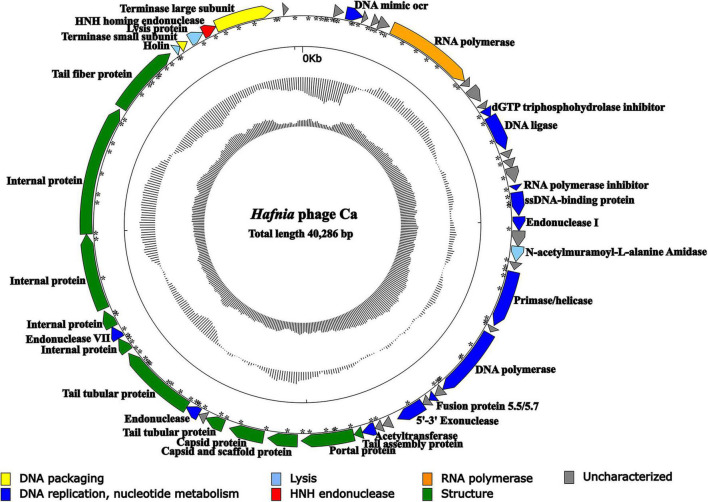
Genomic map of *Hafnia* phage Ca. The direction of the arrow indicates the direction of each gene; the functional groups is distinguished by color: yellow stands for DNA packaging, blue for DNA replication and nucleotide metabolism, azure for lysis, orange for RNA polymerase, green for structure, red for HNH endonuclease, and gray indicates hypothetical protein. Asterisks indicate predicted promoters.

The result of agarose gel electrophoresis indicated that the extracted phage nucleic acid can only be digested by DNase I, but not by RNase A or S1 nuclease, which demonstrated that the nucleic acid extracted from the phage suspension was double-strand DNA (dsDNA) (Supplementary Figure 9). Except for *Hafnia* phage Ca, no other phage genome was found in NGS. The complete genome of *Hafnia* phage Ca is a dsDNA, 40,286 bp in length with a GC content of 50.14%. According to the described method (Zhang et al., 2015), the genomic termini were predicted based on NGS data. *Hafnia* phage Ca has fixed termini with *R* = 230.64>100. The genome of *Hafnia* phage Ca contains direct terminal repeats of 183 bp. Predicted promoters of the *Hafnia* phage Ca genome are listed in Supplementary Table 1 and Figure 4. No tRNA gene was found in *Hafnia* phage Ca. The genomic sequence of *Hafnia* phage Ca was submitted to the Genbank database under accession number MK610268.

The *Hafnia* phage Ca genome contains 50 predicted open reading frames (ORFs); 46 ORFs (92%) have an ATG initiation codon, and 4 ORFs (8%) have a GTG initiation codon. No ORF was found to be associated with virulence factors, or antibiotic resistance genes. *Hafnia* phage Ca is virulent to its host. Correspondingly, no integrase, plasmid-replication gene, transposase, repressor, and genome attachment site were found in its genome, suggesting its good application potential. The genome circular map is shown in Figure 4. All ORFs in *Hafnia* phage Ca genome are oriented in the same direction (Figure 4). Through literature and data search, we found that most *Kayfunavirus* phages also have this characteristic, i.e., their ORFs were predicted to be transcribed from one DNA strand.

The predicted *Hafnia* phage Ca ORFs could be classified into seven functional modules: structure, DNA replication and nucleotide metabolism, DNA packaging, lysis, HNH homing endonuclease, RNA polymerase, and a hypothetical protein (Table 2 and Figure 4). The DNA replication and nucleotide metabolism module of *Hafnia* phage Ca is mainly situated upstream of the structural proteins. While ORF 27 showed 98.96% identity with predicted endonuclease of *Enterococcus* phage EFA-1 (QJT70289) in blastp analysis, ORF 27 was predicted as 5′-3′ exonuclease by RAST, HHpred, and HMMER. Many 5′-3′ exonucleases have been shown to have endonucleolytic activities (Ceska et al., 1996); thus, the endonuclease in *Enterococcus* phage EFA-1 may be an exonuclease possessing endonucleolytic activity. Therefore, ORF 27 was predicted to code 5′-3′ exonuclease in this study. The putative DNA-directed RNA polymerase (ORF 6) was predicted in the *Hafnia* phage Ca, implying that *Hafnia* phage Ca may be primarily dependent on its own RNA polymerase rather than the host RNA polymerase for transcription. The DNA-directed RNA polymerase of *Hafnia* phage Ca shared the highest similarity with that of *Escherichia* phage PE3-1 of *Kayfunavirus* genus, the Studiervirinae subfamily, the Autographiviridae family. A set of structural genes (ORF 33-38) were predicted in the *Hafnia* phage Ca genome. The genes encoding putative DNA packaging terminase of *Hafnia* phage Ca are situated at the end of the genome. *Hafnia* phage Ca harbors multiple lysis genes predicted to encode putative N-acetylmuramoyl-L-alanine amidase (ORF 19), holin (ORF 45), and lysin (ORF 47).

**TABLE 2:** Open reading frame (ORF) analysis of the *Hafnia* phage Ca genome.

ORF	Size(aa)	Prediction function	Top BLAST Hit^a^	Identity^b^(aa)	*E*-value^c^
1	92	Hypothetical protein	ref| QNH91710.1| hypothetical protein [*Escherichia* phage DY1]	93% (86/92)	1e-57
2	169	DNA mimic ocr	ref| AQT25404.1| DNA mimic ocr [*Escherichia* phage vB_EcoP_F]	88% (99/112)	2e-63
3	49	Hypothetical protein	ref| QNJ49171.1| hypothetical protein [*Escherichia* phage Mt1B1_P3]	80% (39/49)	9e-21
4	65	Hypothetical protein	ref| YP_009279733.1| hypothetical protein BI008_gp05 [*Citrobacter* phage SH4]	97% (63/65)	2e-37
5	110	Hypothetical protein	ref| YP_009807448.1| hypothetical protein HOT79_gp01 [*Shigella* phage SFPH2]	94% (101/108)	3e-68
6	893	RNA polymerase	ref| YP_009790620.1| RNA polymerase [*Escherichia* phage ST31]	99% (881/893)	0.0
7	66	Hypothetical protein	ref| YP_009790621.1| hypothetical protein HOR80_gp06 [*Escherichia* phage ST31]	91% (60/66)	1e-33
8	156	Hypothetical protein	ref| YP_002003744.1| gp1.05 [*Enterobacteria* phage EcoDS1]	89% (139/156)	2e-98
9	59	Hypothetical protein	ref| YP_009005118.1| hypothetical protein BN930_gp08 [*Cronobacter* phage Dev2]	98% (58/59)	2e-33
10	94	dGTP triphosphohydrolase inhibitor	ref| QNM37854.1| dGTP triphosphohydrolase inhibitor [*Citrobacter* phage NS1]	95% (83/87)	2e-53
11	358	DNA ligase	ref| QEG09679.1| DNA ligase [*Escherichia* phage Peacock]	93% (331/357)	0.0
12	70	Hypothetical protein	ref| QLF80653.1| hypothetical protein SP7_0010 [*Escherichia* phage vB_EcoP_SP7]	87% (61/70)	4e-37
13	88	Hypothetical protein	ref| QJT70273.1| hypothetical protein EFA_00012 [*Enterococcus* phage EFA-1]	95% (81/85)	7e-53
14	149	Hypothetical protein	ref| YP_009044262.1| hypothetical protein PE3_014 [*Escherichia* phage PE3-1]	92% (97/106)	8e-66
15	52	RNA polymerase inhibitor	ref| YP_009787269.1| host RNA polymerase inhibitor [*Escherichia* phage ZG49]	52/52 (100%)	3e-28
16	232	ssDNA-binding protein	ref| QNJ49158.1| single-stranded DNA-binding protein [*Escherichia* phage Mt1B1_P3]	99% (229/232)	4e-165
17	139	Endonuclease I	ref| YP_009807342.1| endonuclease [*Escherichia* virus Vec13]	99% (137/139)	8e-98
18	156	Hypothetical protein	ref| YP_009044267.1| hypothetical protein PE3_019 [*Escherichia* phage PE3-1]	83% (130/156)	5e-88
19	152	N-acetylmuramoyl-L-alanine amidase	ref| QNH91731.1| N-acetylmuramoyl-l-alanine amidase [*Escherichia* phage DY1]	97% (148/152)	1e-108
20	71	Hypothetical protein	ref| YP_009807345.1| hypothetical protein HOT77_gp22 [*Escherichia* virus Vec13]	99% (70/71)	4e-39
21	566	Primase/helicase	ref| YP_009787275.1| primase/helicase protein [*Escherichia* phage ZG49]	100% (566/566)	0.0
22	54	Hypothetical protein	ref| YP_009044271.1| hypothetical protein PE3_023 [*Escherichia* phage PE3-1]	100% (54/54)	2e-31
23	723	DNA polymerase	ref| AOZ65192.2| DNA polymerase [*Escherichia* phage JSS1]	99% (714/723)	0.0
24	99	Hypothetical protein	ref| YP_009044273.1| hypothetical protein PE3_025 [*Escherichia* phage PE3-1]	90% (89/99)	2e-58
25	69	Fusion protein 5.5/5.7	ref| YP_002003762.1| gp5.7 [*Escherichia* phage EcoDS1]	99% (68/69)	8e-43
26	78	Hypothetical protein	ref| AGD81059.1| hypothetical protein CLBP1_5.8 [*Escherichia* phage CLB_P1]	83% (64/77)	3e-34
27	289	5′-3′Exonuclease	ref| QJT70289.1| endonuclease [*Enterococcus* phage EFA-1]	99% (286/289)	0.0
28	90	Hypothetical protein	ref| QNM37874.1| hypothetical protein [*Citrobacter* phage NS1]	99% (89/90)	1e-58
29	74	Hypothetical protein	ref| YP_009807356.1| hypothetical protein HOT77_gp33 [*Escherichia* virus Vec13]	99% (73/74)	2e-43
30	134	Acetyltransferase	ref| AGD81063.1| hypothetical protein CLBP1_6.8 [*Escherichia* phage CLB_P1]	100% (134/134)	2e-96
31	82	Tail assembly protein	ref| YP_009790642.1| virion assembly protein [*Escherichia* phage ST31]	100% (82/82)	1e-48
32	522	Portal protein	ref| AUX83614.1| head to tail connector [*Escherichia* phage YZ1]	99% (519/522)	0.0
33	294	Capsid and scaffold protein	ref| YP_009044281.1| scaffold protein [*Escherichia* phage PE3-1]	99% (292/294)	0.0
34	347	Capsid protein	ref| YP_009292501.1| major capsid protein [*Citrobacter* phage SH3]	100% (347/347)	0.0
35	188	Tail tubular protein	ref| QEG09809.1| tail tubular protein A [*Escherichia* phage Penshu1]	100% (188/188)	7e-137
36	75	Hypothetical protein	ref| YP_002003774.1| unknown product [*Enterobacteria* phage EcoDS1]	100% (75/75)	4e-48
37	146	Endonuclease	ref| YP_004251224.1| putative endonuclease [*Vibrio* phage ICP2]	59% (47/80)	1e-18
38	785	Tail tubular protein	ref| QOI58480.1| tail tubular protein [*Escherichia* phage vB_EcoP-U8]	98% (770/785)	0.0
39	150	Internal protein	ref| QFP92952.1| internal virion protein [*Escherichia* phage vB_EcoP_PR_Kaz2018]	99% (149/150)	4e-106
40	133	Endonuclease VII	ref| YP_002003777.1| gp13.5 [*Enterobacteria* phage EcoDS1]	95% (127/133)	1e-88
41	175	Internal protein	ref| YP_338124.1| internal virion protein [*Enterobacteria* phage K1F]	97% (162/167)	5e-114
42	760	Internal protein	ref| YP_009798555.1| core protein [*Escherichia* phage YZ1]	99% (754/760)	0.0
43	1295	Internal protein	ref| YP_009007182.1| predicted internal virion protein D [*Citrobacter* phage CR44b]	97% (1259/1295)	0.0
44	726	Tail fiber protein	ref| ARW56875.1| tail fiber protein [*Escherichia* phage ST31]	86% (199/232)	1e-129
45	64	Holin	ref| WP_015978872.1| type II holin [*Escherichia coli*]	100% (64/64)	2e-37
46	87	Terminase small subunit	ref| YP_009324520.1| DNA packaging protein A, T7-like gp18 [*Escherichia* phage LM33_P1]	99% (86/87)	9e-55
47	150	Lysis protein	ref| YP_009818299.1| hypothetical protein HOU92_gp47 [*Escherichia* phage Ro45lw]	93% (135/145)	1e-95
48	143	HNH homing endonuclease	ref| YP_009007189.1| predicted homing endonuclease [*Citrobacter* phage CR44b]	97% (123/127)	1e-85
49	588	Terminase large subunit	ref| YP_009007190.1| predicted DNA packaging protein [*Citrobacter* phage CR44b]	98% (579/588)	0.0
50	52	Hypothetical protein	ref| YP_002003788.1| gp19.5 [*Enterobacteria* phage EcoDS1]	100% (52/52)	2e-27

*^a^ The most closely related protein and its organism. “No hits” indicates no significant hits detected for a particular amino acid sequence.*

*^b^ Percent identity for top hits in BLASTP searches. Numbers in parentheses provide the length of each alignment.*

*^c^ The expected number of hits based on the database size by chance as determined by BLASTP analysis.*

**FIGURE 5:**
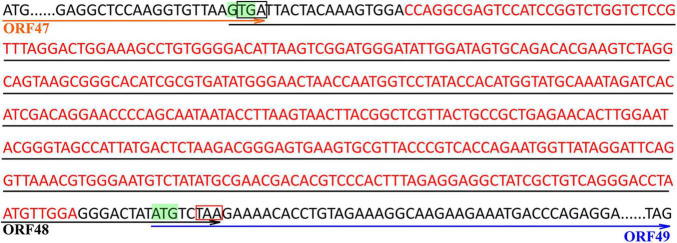
Sequence of the inserted DNA fragment and the adjacent upstream and downstream sequences in the complete genome of *Hafnia* phage Ca. Red letters are the sequence of the inserted DNA fragment. Green GTG and ATG are predicted start codons of the HNH homing endonuclease gene (HEG) and the terminase large subunit gene, respectively. The sequences above the orange arrow are the predicted ORF of lysin (ORF 47). The sequences above the black arrow are the predicted ORF of HEG (ORF 48). The sequences above the blue arrow are the predicted ORF of the terminase large subunit (ORF 49). “TGA” in the black box is the stop codon of the lysin gene. “TAA” in the red box is the stop codon of HEG gene.

Two types of the phage genome, complete and incomplete, were found by high-throughput sequencing. Deletion of a 397-bp fragment containing the predicted HNH homing endonuclease gene (HEG) was found in the incomplete type. So, once again, we sequenced the phage cultures from large, medium, and small size plaques of the sixth generation separately. The relative abundances of complete/incomplete genome types were distinct in large-, small-, and medium-sized plaques. The results showed that the relative abundance of the complete phage genome with the 397-bp fragment was > 98% in the large plaques, while the relative abundance of the incomplete phage genome without the 397-bp fragment was > 98% in the small plaques, and the abundances of them in the medium-size plaques were relatively equivalent (complete, 40%; incomplete, 60%). The upstream and downstream ORFs of the 397-bp inserted fragment were predicted to code putative lysin (ORF 47) and terminase large subunit (ORF 49), respectively, both overlapping with the HEG (ORF 48) (Figure 5). The start codon “GTG” of the putative HEG and the stop codon “TGA” of the putative lysin gene are overlapped (Figure 5). The start codon “ATG” of the terminase large subunit gene was upstream the stop codon “TAA” of HEG (Figure 5).

### Taxonomic Analysis

**FIGURE 6:**
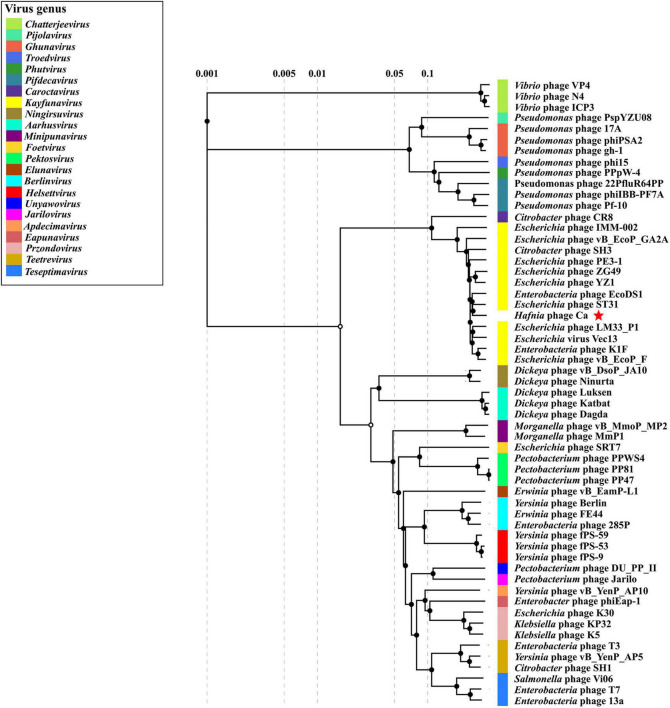
Proteomic tree of *Hafnia* phage Ca and 56 classified phages of the 23 genus of subfamily Studiervirinae of family Autographiviridae. The proteomic tree is generated using ViPTree online based on genome-wide similarities determined by tBLASTx. Bacteriophage family assignments according to the official ICTV classification (2021) are provided with different color bars. The red star indicates phage Ca.

GenBank BLASTN search showed that *Hafnia* phage Ca had the highest sequence similarity (97.02% identity, 86% query coverage) with *Escherichia* phage ST31 (NC_047829.1) of genus *Kayfunavirus* in subfamily Studiervirinae of family Autographiviridae. The ANI and isDDH values between *Hafnia* phage Ca and *Escherichia* phage ST31 were 93.18% and 58.90%, respectively. These values were below the threshold ones for ANI (95%) and DDH (70%) to discriminate viral species, which demonstrate *Hafnia* phage Ca as a novel phage species. The Bacterial and Archaeal Virus Subcommittee (BAVS) currently defined a new genus of viruses with a > 50% of nucleotide sequence similarity (Adriaenssens and Brister, 2017; Adriaenssens et al., 2020). In PASC scanning, *Hafnia* phage Ca shared the highest nucleotide sequence similarity of 82.47% with *Escherichia* phage ST31. In the proteomic tree based on genome-wide sequence similarities (Figure 6), *Hafnia* phage Ca clustered with *Kayfunavirus* phages, especially closely related with *Escherichia* phage ST31 and EcoDS1. Therefore, *Hafnia* phage Ca was classified as a novel species of *Kayfunavirus* genus in the Studiervirinae subfamily of the Autographiviridae family. Autographivirinae was once regarded as a subfamily of the Podoviridae family. In 2019, ICTV removed the Autographvirinae and Autographivirinae-like viruses from the family Podoviridae and assigned a family rank, “Autographiviridae” (Turner et al., 2019). The genomes of Autographivirinae phages are composed of dsDNA of approximately 41 kb with conservative gene arrangement and specific lysis cassettes, and all encode a large (> 100 kDa) single subunit RNA polymerase (Turner et al., 2019).

**FIGURE 7:**
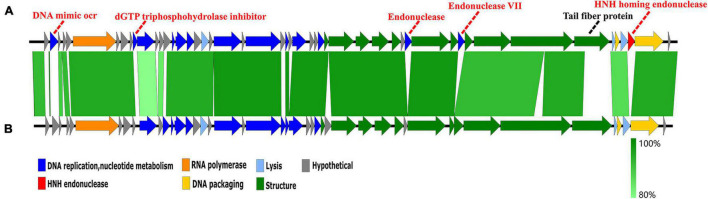
Genome comparison of the *Hafnia* phage Ca **(A)** and *Escherichia* phage ST31 **(B)**. The color of each arrow refers to the functional groups. The orientation of the arrows indicates the direction of gene transcription. The homologous regions are represented by green bars, with their depth reflecting the degree of sequence similarity. Compared to the genome of *Escherichia* phage ST31, *Hafnia* phage Ca genome contains extra genes represented by red dotted lines. The black dotted line indicates a non-conserved gene.

A genome comparison of *Hafnia* phage Ca and *Escherichia* phage ST31 is shown in Figure 7. *Hafnia* phage Ca shares 38 ORFs with *Escherichia* phage ST31. The arrangements of these homologous ORFs are essentially the same. While the N-terminus of tail fiber protein of *Hafnia* phage Ca (ORF 44) exhibited 85.78% identity to the N-terminus of tail fiber protein of *Escherichia* phage ST31, their C-termini were not conserved. Previous studies have shown that the changes in the C-terminus of the tail fiber protein contribute to changing the host specificity of the phages (Tétart et al., 1996; Le et al., 2013; De Leeuw et al., 2020). Compared to the genome of *Escherichia* phage ST31, *Hafnia* phage Ca genome contains extra genes including DNA mimic ocr (ORF 2), dGTP triphosphohydrolase inhibitor (ORF 10), endonuclease (ORF 37), endonuclease VII (ORF 40), and HNH homing endonuclease gene (ORF 48) located between the terminase large subunit and small subunit (Figure 7).

### Protective Effects of *Hafnia* Phage Ca in Brocade Carp

In the experiments under the condition without aeration, the cumulative mortality of the blank, control, and test groups was 5.8, 94.4, and 64.2%, respectively. The protection rate was 31.99% (*p* < 0.01).

In the experiments under the condition with continuous aeration, the daily number of deaths and cumulative mortality of brocade carp in the test groups, control groups, and blank groups are listed in Supplementary Table 2. The cumulative mortality of brocade carps in the test groups was 46.67% ± 9.43%, which was significantly lower than those in the control groups, of which the cumulative mortality was 80.00% ± 8.16%. The protection rate was 33.38% (0.01 < *p* < 0.05).

Aeration had an effect on the death of brocade carps and the protection rate. Death occurred within 5 days under the condition without aeration and occurred with in 7 days under the condition with continuous aeration. Under conditions with continuous aeration, the mortality of each group was much lower than that of the corresponding group. The protection rate under the condition with continuous aeration was 33.38%, which is slightly higher than that (31.99%) under the condition without aeration. The data indicated that *Hafnia* phage Ca has an apparent protective effect on brocade carps.

## Discussion

*Hafnia* was considered to be an opportunistic pathogen to a variety of animal species including fish, mammals, and birds, and is suspected to cause human infection (Huys et al., 2010; Osuka et al., 2011; Padilla et al., 2013). In this study, an antibiotic susceptibility test demonstrated that *H. paralvei* LY-23 was multidrug-resistant. Phages are used as a potential alternative and effective treatment for diseases caused by multidrug-resistant bacteria pathogens. However, although six *Hafnia* phage genomes can be found in GenBank database, *Hafnia* phage has never been studied systematically. No information about plaque morphology, phage morphology, host range, temperature stability, pH stability, chloroform sensitivity, one-step growth curve, and therapeutic application of *Hafnia* phage can be found in the current literature. In this study, *Hafnia* phage Ca was isolated and identified as a novel phage. *Hafnia* phage Ca was classified as a novel species of *Kayfunavirus* in the Studiervirinae subfamily of the Autographiviridae family in consideration of the proteomic tree, pair-wise ANI, isDDH, and PASC values and conformed to its morphological characteristics.

The therapeutic value of a candidate phage depends on the characterization of phage properties such as stability, growth kinetics, host range, and viral yield (Jacquemot et al., 2018). Genome sequencing and analysis is also vital for assessing the safety of a candidate (Lobocka et al., 2014). The traits and genomic analysis of *Hafnia* phage Ca indicated that *Hafnia* phage Ca is a good candidate for control of *H. paralvei* LY-23. Firstly, the results of this study showed that its temperature and pH stabilities were good, which is to the benefit of control of *H. paralvei* infections in a complex environment. Second, its latent period (≤ 12 min) is short. Thirdly, it only infects and lyses *H. paralvei* LY-23 and *A. sobria* ATCC43979 of the strains tested. In addition, results also demonstrated other good properties including a low optimal MOI (0.001) and abnormally high burst size. The burst sizes of phage Ca against *H. paralvei* LY-23 at MOI of 0.01 based on one-step growth curve and qPCR were similar, which are 10,292 ± 1,097 PFU/cell and 14,708 ± 1,534 copies/cell, respectively. It is also very high in *A. sobria* ATCC43979, which is 11,766 ± 1,033 PFU/cell. Finally, no ORF was found to be associated with virulence factors, or antibiotic resistance genes in the *Hafnia* phage Ca genome. Results of protective effects of *Hafnia* phage Ca in brocade carp showed that *Hafnia* phage Ca has an apparent protective effect. According to the above results of the study, *Hafnia* phage Ca is considered to have potential application value.

In this study, in the one-step growth experiments under the MOI of 0.001, total phage titers and free phage titers in the samples collected at various time points were counted. The former was used to plot the one-step growth curve. The curve based on the latter, verifying the one-step growth curve, was also valuable. Therefore, we promote that both the total phage titers and free phage titers should be detected and both the curves should be drawn in the study on proliferation kinetics of phage.

Results of the host range test showed that *Hafnia* phage Ca could lyse *H. paralvei* LY-23 (belonging to Enterobacterales) and *A. sobria* ATCC43979 (belonging to Aeromonadales). This is unexpected as most reported phages are specific to the host strain. Nevertheless, although a narrow host range is prevalent among most isolated and well-studied phages, more and more broad-host phages were found recently. It was proposed that broad host ranges may be more prevalent than previously considered (De Jong et al., 2019; Lin et al., 2020). Phage VP882 was found to infect some bacterial strains of Vibrionaceae and Enterobacteriaceae (Silpe and Bassler, 2019); Cyanophage vB_MelS-Me-ZS1 was found to lyse some cyanobacterial strains of three orders including Chroococcales, Nostocales, and Oscillatoriales (Lin et al., 2020). Aquamicrobium phage P14 was found to infect two different Aquamicrobium strains belonging to the Alphaproteobacteria class and three different Alcaligenaceae strains belonging to the Betaproteobacteria class (De Leeuw et al., 2017). The coming to light of more and more phages having broad host ranges may be the result of phage isolation techniques, host range experimental approaches, the trade-off between host range and phage growth rate, and the virulence on each individual host (De Jong et al., 2019; Lin et al., 2020). Molecular mechanisms of phages’ host range are complex and involved in every phase of the phage life cycle. The mechanism of *Hafnia* phage Ca host range across two orders is worth pursuing. The EOP value of *Hafnia* phage Ca was 0.82 ± 0.11. The phage yield in *H. paralvei* LY-23 (1.42 × 10^11^ ± 1.5 × 10^10^) was higher than *A. sobria* ATCC43979 (3.58 × 10^10^ ± 2.60 × 10^9^) in the one-step growth curves, while the calculated burst size of *Hafnia* phage Ca in *A. sobria* ATCC43979 was slightly higher than that in *H. paralvei* LY-23. This contradiction is caused by the denominators, i.e., higher adsorption-infection rate and more infected cells of *H. paralvei* LY-23.

An interesting phenomenon was discovered that the phage *Hafnia* phage Ca developed at least three obviously different sizes of plaques when a single plaque was picked out and inoculated on a double-layer Luria broth agar plate with its host. A similar phenomenon had only been found to be previously described on *Stenotrophomonas* phage IME13 of *Tulanevirus* genus of the Myoviridae family (Fan et al., 2012). The NGS-based analysis demonstrated that the plaques of different sizes of *Hafnia* phage Ca contain complete and incomplete genomes with different ratios. We propose that, regardless of the size, all plaques are formed by the propagation of the phages with complete genomes, but other non-infectious virions with a 397-bp deletion in the HEG gene are also present in the plaques. Furthermore, variable tail packaging tightness of *Hafnia* phage Ca was observed. The incomplete type of genome of phage Ca with a deletion of 397-bp fragment codes putative HNH homing endonuclease. The upstream and downstream ORFs near the 397-bp inserted fragment were predicted to code lysin and the terminase large subunits, respectively, both overlapping with HEG. Some phage genomes possess genes coding HNH protein (protein-containing HNH motif) near their terminase genes (Moodley et al., 2012; Kala et al., 2014). It was identified and reported that HNH endonucleases (gp74) are required for the specific endonuclease activity of the phage HK97 terminase and are essential for the head morphogenesis of the phage (Kala et al., 2014). Bioinformatic analysis of Kala et al. (2014) revealed that the role of HNH proteins in terminase function is widespread among long-tailed phages and is uniquely required for the activity of the Terminase1 family of large terminase proteins. HEGs, encoding endonucleases capable of cutting site-specific DNA that promotes the lateral transfer of their own, can themselves be mobile elements moving independently (Mota and Collins, 1988; Stoddard, 2005; Buttimer et al., 2020; Dutheil et al., 2020). Mobile genetic elements have the potential to influence the expression of genes surrounding their insertion site upon invasion of a genome (Gibb and Edgell, 2007). We speculate on the following possibilities: the mobile 397-bp fragment, harboring HEG near the terminase gene and lysin gene, may be conducive to the expression of the terminase (involving in phage package) and lysin (involving in bacteriolysis and phage release) of *Hafnia* phage Ca. If the expression level of lysin gene is affected, it may lead to the change of bacteriolytic efficiency and affect the size of plaque; if the expression level of the terminal large subunit gene is affected, it may lead to the change of packaging efficiency and affect the morphology of phage. This matter is worthy of further research. HEG was also found in the genome of *Stenotrophomonas* phage IME13, which also has a huge burst size of 3,000 PFU/cell and always developed variable size of plaques (at least three obviously different sizes) when a single plaque was picked out (Fan et al., 2012).

The burst size of most reported phages, including Autographiviridae phages, was at dozens to hundreds of PFU/cell (Baudoux et al., 2012; Huang et al., 2013; Merabishvili et al., 2014; Qin et al., 2017; Anand et al., 2018; Lu et al., 2020). A literature review found *Stenotrophomonas* phage IME13 of the Myoviridae family and *Cronobacter* phage vB_CtuP_B1 of genus *Kayfunavirus* in subfamily Studiervirinae of family Autographiviridae had especially prominently high burst size of 3,000 (Fan et al., 2012) and 4,006 PFU/cell (Zeng et al., 2021), respectively, which were the highest reported burst sizes of dsDNA phages. The burst size of *Hafnia* phage Ca was much larger compared with other dsDNA phages including *Kayfunavirus* phages. Such a big burst size had not yet been reported among dsDNA phages. The burst size of phage is not limited by cell size or DNA composition, but rather related to the rates of synthesis and assembly of phage components, latent period, host metabolic activity, survival environment, and protein-synthesizing machinery of the host bacteria (Hadas et al., 1997; Wang et al., 2019; Sui et al., 2021). The latency (2–12 min) to rise period time (about 110 min) ratios of *Hafnia* phage Ca were unusual. This may have something to do with heterogeneity in latent periods. The molecular mechanism of the abnormally large burst size of *Hafnia* phage Ca needs further study.

Compared to the genome of the closest related *Escherichia* phage ST31, the *Hafnia* phage Ca genome contains extra genes including the putative DNA mimic ocr, dGTP triphosphohydrolase inhibitor, endonuclease, endonuclease VII, and HNH homing endonuclease gene, which may contribute to its unusual high burst size. DNA micic ocr is an inhibitor of the complex type I DNA restriction enzymes by preventing them from binding to their DNA target (Walkinshaw et al., 2002). *Hafnia* phage Ca may use DNA micic ocr as a shield for breaching the bacterial defense provided by the type I DNA restriction/modification (R/M) system. dGTP triphosphohydrolase inhibitor inhibits dGTPase activity by forming a complex (Singh et al., 2015), which may constitute another shield for *Hafnia* phage Ca to defend the host. Endonuclease, endonuclease VII, and HNH homing endonuclease capable of cutting host DNA may participate in the attack on the host as *Hafnia* phage Ca’s spears. Viruses and their hosts are often portrayed as a biological arms race. These excess “spears and shields” in *Hafnia* phage Ca may be associated with its enormous burst size.

In a word, the biological and genomic characteristics of the novel *Hafnia* phage Ca is unique. It develops different sizes of plaque and has variable packaging tightness of tail and very huge burst size. Are the characteristics of Ca related to two types of phage genome (complete and incomplete genome)? The specific molecular mechanism of these characteristics needs to be further studied.

## Data Availability Statement

The datasets presented in this study can be found in online repositories. The names of the repository/repositories and accession number(s) can be found in the article/Supplementary Material.

## Ethics Statement

The animal study was reviewed and approved by Ethics Committee for Animal Experiments of School of Marine Sciences of Ningbo University.

## Author Contributions

DL, LP, ZS, and YT designed the research. LP, ZS, WQ, BH, LX, WLi, QZ, FW, RC, MQ, and DL performed the experiments and interpreted the data. LP and DL wrote the manuscript. All authors contributed to the article and approved the submitted version.

## Conflict of Interest

The authors declare that the research was conducted in the absence of any commercial or financial relationships that could be construed as a potential conflict of interest.

## Publisher’s Note

All claims expressed in this article are solely those of the authors and do not necessarily represent those of their affiliated organizations, or those of the publisher, the editors and the reviewers. Any product that may be evaluated in this article, or claim that may be made by its manufacturer, is not guaranteed or endorsed by the publisher.

## Footnotes

1
http://www.softberry.com/berry.phtml

2
https://rast.nmpdr.org/rast.cgi

3
https://www.ebi.ac.uk/Tools/hmmer/search/hmmscan

4
https://toolkit.tuebingen.mpg.de/tools/hhpred

5
http://lowelab.ucsc.edu/tRNAscan-SE/

6
http://www.ezbiocloud.net/ezgenome/ani

7
http://ggdc.dsmz.de

8
http://www.ncbi.nlm.nih.gov/sutils/pasc/

9
https://www.genome.jp/viptree/
