# Supplementary material for: First Characterization of a Hafnia Phage Reveals Extraordinarily Large Burst Size and Unusual Plaque Polymorphism
Source: Front Microbiol. 2022 Feb 8;12:754331. doi: 10.3389/fmicb.2021.754331 (PMC8861465; doi:10.3389/fmicb.2021.754331)
Supplement: Supplementary file 1 [file Data_Sheet_1.docx]

**SUPPLEMENTARY MATERIALS**

**Figure S1.** Results of antibiotic susceptibility test of *H. paralvei* LY-23. The results showed that *H. paralvei* LY-23 was resistant to 7 of 17 tested antibiotics, which were cephalexin, penicillin G, amoxicillin, aboren, clindamycin, vancomycin, and rifampicin. Its sensitivity was intermediate to kanamycin, azithromycin, and doxycycline.

**Figure S2.** The one-step growth curve of *Hafnia* phage Ca against *A. sobria* ATCC43979 showed the latent period was 2–12 min and its burst size was 11,766 ± 1,033 PFU/cell.

**Figure S3.** The final phage titers of phage Ca under MOIs of 0.0001, 0.001, 0.01 , 0.1, 1, and 5.

**Figure S4.** The adsorption kinetics of *Hafnia* phage Ca. Adsorption of Ca to *H. paralvei* LY-23 is very efficient, and the adsorption rate can reach 97.60% ± 0.23% in 2 min at 29℃.

**Figure S5.** Standard curve obtained by 10-fold dilution series of the positive standard phage DNA. Quantitative fluorescent PCR detection on phage DNA standards resulted in the standard curve formula: *y* = −2.346*x* + 28.46 (*R*^2^ > 0.99, the closer the *R* squared value gets to 1, the more reliable the standard curve).

**Figure S6.** The one-step growth curve of *Hafnia* phage Ca under the MOI of 1. The latent period of *Hafnia* phage Ca was 2–12 min. The burst size of phage Ca at MOI of 1 was 8,605 ± 244 PFU/cell.

**Figure S7.** The one-step growth curve of *Hafnia* phage Ca under the MOI of 5. The latent period of *Hafnia* phage Ca was 2–12 min. The burst size of phage Ca at MOI of 5 was 3,713 ± 130 PFU/cell.

**Figure S8.** The titer variation curve of free virions of *Hafnia* phage Ca under the MOI of 0.001.

**Figure S9.** [Agarose gel electrophoretogram of](https://image.baidu.com/search/detail?ct=503316480&z=&tn=baiduimagedetail&ipn=d&word=electrophoretogram%201%25%20agarose%20gel%20electrophoresis&step_word=&ie=utf-8&in=&cl=2&lm=-1&st=-1&hd=&latest=&copyright=&cs=3549915188,3269470975&os=730306023,1362547627&simid=3549915188,3269470975&pn=9&rn=1&di=23880&ln=305&fr=&fmq=1635650990106_R&ic=&s=undefined&se=&sme=&tab=0&width=&height=&face=undefined&is=0,0&istype=2&ist=&jit=&bdtype=15&spn=0&pi=0&gsm=0&objurl=https%3A%2F%2Fgimg2.baidu.com%2Fimage_search%2Fsrc%3Dhttp%253A%252F%252Fwww.spandidos-publications.com%252Farticle_images%252Fol%252F14%252F2%252Fol-14-02-1427-g00.jpg%26refer%3Dhttp%253A%252F%252Fwww.spandidos-publications.com%26app%3D2002%26size%3Df9999%2C10000%26q%3Da80%26n%3D0%26g%3D0n%26fmt%3Djpeg%3Fsec%3D1638242988%26t%3Dc3158ebfdb5605245970b37be39d8035&rpstart=0&rpnum=0&adpicid=0&nojc=undefined&dyTabStr=MCw1LDEsNiw0LDMsNyw4LDIsOQ%3D%3D) the nucleic acids of *Hafnia* phage Ca untreated (lane 1) or treated with DNase I (lane 2), RNase A (lane 3), and S1 nuclease (lane 4). To clarify the type and composition of the nucleic acid extracted from the phage suspension, the nucleic acid was treated with DNase I, RNase A, and S1 nuclease at 37℃ for 30 min, respectively, and detected by 1% agarose gel electrophoresis. The result indicated that the extracted phage nucleic acid can only be digested by DNase I, but not by RNase A or S1 nuclease, which demonstrated that the nucleic acid extracted from the phage suspension was dsDNA.

**TABLE S1.** Predicted promoters in *Hafnia* phage Ca genome

| Start | End | Score | Promoter sequence |
| --- | --- | --- | --- |
| 160 | 205 | 0.95 | CTATTGGTCTGTCTCTGAGTCACTATCTGTATAATGATATAGTATAACAT |
| 430 | 475 | 0.89 | TATTTCAAATTACCTATTGACTATAGGTCTAGACTATGGCTTAATAGCTT |
| 582 | 627 | 0.97 | GGTAGTTGACAAGTAGCACCGACTTAAAGTAGTATGTACCACGTAGCAAG |
| 691 | 736 | 0.93 | CCGTGAGTCGAATTCGGTCACTCACCGCATAAAAGAATAATAGGTTGACA |
| 729 | 774 | 0.92 | AATAGGTTGACAGCCTCCTTTAAAAGCTGTAAGCTGTACCACATCGAAAC |
| 912 | 957 | 0.81 | CTGATAGTCACTAACAACCTAAGAGGATTCATTATGAGACTGAACAAAGC |
| 1003 | 1048 | 0.83 | TGCGTGGAGTGGCGGCGGAAGCCAAGCGTTATAAAGAGAACGGCCTAACG |
| 1065 | 1110 | 0.83 | ATGTGGGATTGCTACCATGGAGTGGGCCATAAAAAGTCGCAGGAAATAAT |
| 1516 | 1561 | 0.86 | CTGATGCCTGAGACCAAGGACGTAACGCGTATACTACAAGCTCGCATCTA |
| 1559 | 1604 | 0.81 | GCATCTATGAGACGCTGTATAACGACGTGTCTAATAGCTCGGATGTGGTT |
| 1741 | 1786 | 0.89 | CATCACATGAAAGTCGAAGACATCAACGATAACGTAGTGTTCGACCCTGC |
| 2044 | 2089 | 0.97 | TATTTGGTATGCCTAGCGCCCGTTATCTGGATGATAGGTTATGGGATATA |
| 2166 | 2211 | 0.98 | GTGCTGGTGAAAGACATCAACAAAGCGCGTAAAGTCTACAAATTCAACTA |
| 2295 | 2340 | 0.81 | ACACTTGGGAGCAAACTGTAATGAGTAAATCTTATGGCATCAACTTGGTC |
| 2504 | 2549 | 0.94 | TATGGTGGACGACCAATCACTGACGACGGTAAACTTCTGGTCTCAGGCTG |
| 2625 | 2670 | 0.96 | CATTGATATGCAAGCAATTCTGACTTAACTATCACTATAGGACTCAAGGT |
| 3204 | 3249 | 0.90 | CGCACACTTGAAGATGAAATCCGCTTCGGTCGAATCCGAGACAAGGAGCA |
| 3708 | 3753 | 0.90 | TACATGCCTGAAGTCTATGACGCTGTGAACATCATCCAGAATACACCTTG |
| 4130 | 4175 | 0.96 | TGGTTTTAAATGGCTGAAGGTCCACGGTGCAAACTGTGCGGGTGTTGATA |
| 4484 | 4529 | 0.98 | CATTGTGTCTGACCGCATCGAGGAGGAGCTTAAATCCCTGCTGGTTAACG |
| 5343 | 5388 | 0.96 | GACAAGTTGCCTGAGCTTCCGAAGAAAGGTAAACTGAATATCGAAGACAT |
| 5405 | 5450 | 0.82 | TTTTGCATTCGCTTAACAGGAGAGAGTTATGAAATTTGCACACAAGCAGA |
| 6576 | 6621 | 0.92 | ATGAGACTTCAAGTCGAGGACCGAGCTGGTAACATCTTCGCGCTTGAGAT |
| 6809 | 6854 | 0.94 | AAGCTGAAGTGAAGTACGATGGTGTCCGGTTGAATCTTCCGGTGCTGCGA |
| 7405 | 7450 | 0.80 | TACAAGCGTGGCAAGAAGTCGGGCATGTGGAAAATGAAGCCTGAGGAGAC |
| 7955 | 8000 | 0.83 | CTATGAATGGCGTGAAGCCTGCAGAACTGTAGCTTCTCTTGAGGCGGCAG |
| 8172 | 8217 | 0.84 | CGCTTGCATTAAGCGTAACGAGAAGCTGGGATACTTCGGGAAGGCCGTTA |
| 8600 | 8645 | 0.92 | CAGGTCTTTGACGGTGTGGAGTCCATTGAGATTATAGCCCGGTCCATGAC |
| 8970 | 9015 | 0.80 | CTCTTGAAGAGGCTACACTGAAGTCCCAAGAGTATGAGGACGCTGGGTTT |
| 9192 | 9237 | 0.91 | AAGTTTAACGAGCGTGGTGAGCATAAGGTTAACCTGACATTCGACCTGAG |
| 9395 | 9440 | 0.86 | GAATGGTGATGGTACTGTTACCCTGAAGTTTAAATGCTTCGCGTCTTACC |
| 9438 | 9483 | 0.87 | TCTTACCTGAAGGATGGTAAGTCAGAGCCTATCGTATTACGGTTCTACGA |
| 10196 | 10241 | 0.92 | AGAGAGGTGACTGTACGTCTGCCTTCAGGCATACTCATTCCCAAGAAGAA |
| 11148 | 11193 | 0.92 | ACGAACTGGTCACTTCCGACCACGGTTAATAAATTCTAAAGGAGAACAAC |
| 11390 | 11435 | 0.85 | CAGTTGGCTCAGTTCTTCAAGGACTAAACTATCACCTTAGGGATGGGACC |
| 11462 | 11507 | 0.99 | CATTTGTGATTAAGGAGTGACCAATGTCATACGATGACCGAGACGACGAG |
| 11664 | 11709 | 0.82 | AGGAGGTAGAAAGCAAGTGAGCAATCTGTTAACGTTCGGTGAGAACGCTG |
| 12061 | 12106 | 0.86 | AACCTTGAGTATCTCGACCAGTTCGAAGAGATAATCTTGATGTTCGACAT |
| 13121 | 13166 | 0.87 | CGATTGGCTTCACTGGCAGCAGCGGAGAAGAGGATAGCGGCTCGTGGGAA |
| 13200 | 13245 | 0.98 | ATTGTGACTGGAGGTCGCCATATGGAGGACATCATTAGTGTTAAAGAAAC |
| 13232 | 13277 | 0.98 | CATTAGTGTTAAAGAAACTTAAAGCTCGCTACCATCGGTTCATGTACAAA |
| 14045 | 14090 | 0.90 | CGTTGTTCAAGAAGTTCCTGACTGACAAGTATTACTTCCAGTCTGAACAG |
| 14541 | 14586 | 0.81 | GGACTTCACTGACACCGGGAAACCTGTAGTAGATGACGAGACGTTAGAAC |
| 14566 | 14611 | 0.92 | GTAGTAGATGACGAGACGTTAGAACACGTTAAGTTACCAGACGCAGAGGC |
| 15482 | 15527 | 0.87 | ACGAATTGCAGATTGCGTGTCGTACTCAGGAGATTGCCGAAGAAGTCGTC |
| 15542 | 15587 | 0.84 | AACTTGCTATGCGTAAGGTGGGTGAGTTCTATAACTTTAAATGTGTCCTT |
| 15574 | 15619 | 0.93 | AACTTTAAATGTGTCCTTGATACCGAAGGTAAGATTGGACCAACGTGGAA |
| 15705 | 15750 | 0.98 | TTAATTGTTAATGTTCTGCGAGAGCTGGCTAAAGTCGTTCACTCAGGTGA |
| 16709 | 16754 | 0.86 | CCTACGCTTGAGGGTGATGACTGTATGGGTATCATCGGGACCCGACCTCA |
| 17830 | 17875 | 0.88 | TCGGATGTGGACACCAACGAGACAAAAGGTATCAAAGACCTGAAGGTCAA |
| 17999 | 18044 | 0.93 | GCTTGTCGCCTCTGGGAAGACGGGCAGTCTAACTTCAAATCATTCGAGGA |
| 18635 | 18680 | 0.86 | GCTGCTGAAGGCGCGAAGTCAGTTTATGATAGATTGAAGAACGGACGACA |
| 19106 | 19151 | 0.82 | TACGTTGTTCAACGTGATGCTTTCGGTAATATCTTGCAGATTGTGACTCT |
| 19140 | 19185 | 0.94 | TGCAGATTGTGACTCTCGACAAGGTAGCGTTTAGTGCTCTACCTGAAGAC |
| 19430 | 19475 | 0.90 | TCGCTGGAGACGATTACAGAAGCTATCACCAAAATGGCTAAGGTAGCCTC |
| 19907 | 19952 | 0.96 | CAGGACTTGGAGAAGCTGACTCAGGCGGTTAACATGATGACTGGTCTTCA |
| 20156 | 20201 | 0.92 | GCTGGTGAGGACATGGCTCAAGCCTAAACTATCACTATAGGAACACCGAA |
| 20627 | 20672 | 0.93 | TGCATTTGACGACATGGTGTCTTCTGCTGTAGAAGCTGGCAAGGTCACTG |
| 21250 | 21295 | 0.92 | GGATTGGGCATACAGTAAGTAATAAACTTTATCTTTCAATTGAATAGGAG |
| 22068 | 22113 | 0.90 | GGTGCTGGTGAGACCCGTGGTGAAGATGGTATCACTATCGCTTCCGGTCA |
| 22527 | 22572 | 0.92 | CTTAAAGTGAGAGGAGACTTATGGCTCAATACATTCCACTGAATGCTAAC |
| 22739 | 22784 | 0.95 | GCTGTCCTGACACCTGATGTCCAAGACAATAGGATTCGATTCTTGCCGTC |
| 23293 | 23338 | 0.81 | GACGTTGGCGACATCGCTGAAGGATACGTCCTAATGAGTATGATTTAAGT |
| 23503 | 23548 | 0.90 | AAGACTGGAAGAAGAACAGTCAGCTTAAATACCTTTATGGCATCGACCTA |
| 23535 | 23580 | 0.82 | CTTTATGGCATCGACCTAGAGAGGTTCAATGAAATGCGAAAGGAGGCAAA |
| 23557 | 23602 | 0.95 | GGTTCAATGAAATGCGAAAGGAGGCAAACTATCAGTGCCAAATCTGTGGG |
| 23604 | 23649 | 0.87 | GGGTGTCATGAGAGCGAGAGTACAGGAGGGACATTATGTGTTGACCACAA |
| 23798 | 23843 | 0.90 | CTTAAAGGTGGCATTAGCCAGCAGCCTGATATACTGAGGTTCTCAGACCA |
| 24159 | 24204 | 0.97 | AAGGTGGAAACGAGAAGTCACACTCTGGTTATAACCGCAAAGCACGAGCT |
| 24562 | 24607 | 0.84 | TCAAGGTGAGACAAACAGTAGCGCCGATGAATACTACGTGATGTATGACT |
| 24644 | 24689 | 0.83 | CCGGGAGTTGTCACTGGGTTCGATAACACCACAATGCCGCACGCTCTGGT |
| 25496 | 25541 | 0.83 | GAGGCAACTGACTTCCAGTCAGAGCCATATAGACTACACATTGACTCCAA |
| 25523 | 25568 | 0.91 | TATAGACTACACATTGACTCCAAGGTGTCTATGGTAATTCCAATTGGCTC |
| 25560 | 25605 | 0.93 | TTCCAATTGGCTCATATAATGCTGACACATATAAGACTACGGTTGACATT |
| 25688 | 25733 | 0.96 | GGGGACTTGACCAGTAGCTCTAATGTGGTTATCCTCAACGGCGATTGGTC |
| 25979 | 26024 | 0.90 | AACATTGGTGACGGGCAGTATCGCTTCGCTATGAATGGTAACGCATTAAC |
| 26128 | 26173 | 0.85 | TATTGAAGGGCCTATAGATTTACCTTAACTATCACTATAGGGACTATAGG |
| 26371 | 26416 | 0.94 | AAGTGTTTGCCTATGGTGGAAACCAAGGGGATAACGTATGGTTCTTGACT |
| 26526 | 26571 | 0.92 | TACGTGTGGTCAGGCAATAAGTCTCACATTAAATTCTTGAAGTTGCTTGG |
| 26560 | 26605 | 0.97 | TCTTGAAGTTGCTTGGGGCTAAGTTCCATGATGATTGGACTATCAGCCCA |
| 26825 | 26870 | 0.93 | TGCTATGGAATAACACCTAAGGAGTATGATAAAATGCTTCGTGAGCAAGG |
| 27339 | 27384 | 0.93 | GACAGGATTGAACGTCAGGTCAAAGGAGATACAGTCAAGGAGCGAGCAGG |
| 27393 | 27438 | 0.83 | ACCGAAAGTTACAACCGCGACTATGCGGCTATCTTCGGGAACCGTATCGC |
| 28518 | 28563 | 0.95 | GCTATGGTTGAGGCCCAAGGTACTGAGTATAAACTGGTGGCTAAGTATCA |
| 29772 | 29817 | 0.86 | GCAGAACGTGACGCTAACAAGCGAGCGCGTATCGTCGGGACTAAAGCTGC |
| 29940 | 29985 | 0.89 | TCTCGTGGAGTGCGCAATAATAATCCGGGTAACATCCGTAAGTCTAAGGA |
| 30909 | 30954 | 0.88 | CAGGTTGGTAAAGGGGGTAAGCTGGTCAACAAGATGTTCACCGTGGCTGC |
| 31137 | 31182 | 0.88 | ACACGTCTGGAAGCTCGTGAGACTGCTCGTAACGTTGATGGTCAGGACCT |
| 31788 | 31833 | 0.89 | GCGATGTTTGGTCGTCCAGATGCTCAGTCTATCTTTCCGGGTAGCCGCTT |
| 32529 | 32574 | 0.80 | GGTGTTCAGAACTTAACGGAGATTGGTGGTATGCTAGCTCGTGGTAACGT |
| 32799 | 32844 | 0.94 | GGTGAACTTGCAGTACGCTCTCCGTTCACTAAAGTCCTCAACGGTACGAC |
| 33468 | 33513 | 0.91 | TTGGGTGGCATCGCTGGGTATGAGGACACTAAGATTCTCCGCTCGTCTAT |
| 33604 | 33649 | 0.95 | ACTTCTTGGAGCAGGTTCCAGCTTTCGGATATGCTGCTAACGTTGGCGCT |
| 34577 | 34622 | 0.86 | AATTGAATTTATCATCAGTAGGGACTGCATCAATTCAAGGAGTTCATGAG |
| 34957 | 35002 | 0.98 | ATTTTTGAAAACGTGCAGGTGTTTAATACCAAATTTGGTGTGCGACTTGG |
| 35059 | 35104 | 0.93 | CAAATGTCTGGAACCAGTACGCACTATGATAACGTATGGGCACGTGGCCA |
| 35299 | 35344 | 0.88 | TTTGATACTGGCAACAGAATTACTCTTAATAACTTCCAGATTAGTCCACT |
| 35389 | 35434 | 0.95 | CCTTTGATTACTGTGGGCAATGATAACAACATCATCATCGAACGTCTGTT |
| 35690 | 35735 | 0.91 | TAATTGGTGGTTCTAGTATTGGTATTACTTATAGTGCTCGCTCTGGTACA |
| 35903 | 35948 | 0.90 | TACTTGGTTCAGTATTAGCTGGAGACAACTCTATTCCATTGCGCAGAGTA |
| 36186 | 36231 | 0.81 | AACAACTTTGGGTCACGGACGACCCGCTTTAAACTGGAGGTTTTATGATT |
| 36211 | 36256 | 0.90 | GCTTTAAACTGGAGGTTTTATGATTGAGTTAGACTTCAAGAATGAGGTCC |
| 37080 | 37125 | 0.82 | TCAGATGTCAGTCGATGCTTCCCTAATGGTAGAGTCGAACTACACCCAGA |
| 37506 | 37551 | 0.83 | AGACGGGAGTGAAGTGCGTTACCCGTCACCAGAATGGTTATAGGATTCAG |
| 37842 | 37887 | 0.93 | TCGTTTTTAACCTGTGGTTTCGTGGTCTGGAAACTATGGAACAACCCAGA |
| 38271 | 38316 | 0.92 | CGTGAGCTGGAAGGTCGTGGGTACACCACTACCATCTGGCCTGCTCGTTA |
| 38746 | 38791 | 0.86 | GTAAGGATGAAACTGGTTATGCGGTACTGTATCAGCTCAACGGCTACATC |
| 39432 | 39477 | 0.84 | AAAGCTGCATGAATACGCAGTTAGTAACCTATAGTTACTACCAGTCTAAC |
| 39483 | 39528 | 0.99 | TACTGTTTTACAAGGAGTTTGGACTTAACTATCACTATAGGGAAGACCCC |
| 40031 | 40076 | 0.89 | ACGGTCCTTGACCTACAGTGGCTGAGGCATACAGTAGGGACGATGAGTTT |

The underlined letters are TATA boxes and Sextama boxes of predicted promoters.

**TABLE S2.** The daily number of deaths and cumulative mortality of the brocade carps of the test groups, control groups, and blank groups in [the therapy experiment](https://www.baidu.com/link?url=djSOpdlhewl1f32yVjDfbL3pxTTj3rXL0xEAtKUrUZ438jHEUoeqXAV0FZASXt9306j2ftlX5h_AYL7RNkbCi_WBkw8dl5VwASD7EyRemmOEkeL7WZFqBwSne2f8DeZf&wd=&eqid=ab573d460000112000000004614ad21e)with continuous aeration.

| Day | The daily number of death and cumulative mortality of the test groups treated with *H. paralvei* and phage Ca | | | The daily number of death and cumulative mortality of the control groups treated with *H. paralvei* | | | The daily number of death and cumulative mortality of the blank groups | | |
| --- | --- | --- | --- | --- | --- | --- | --- | --- | --- |
|  | Group 1 | Group 2 | Group 3 | Group 1 | Group 2 | Group 3 | Group 1 | Group 2 | Group 3 |
| 1 | 0 (0%) | 0 (0%) | 0 (0%) | 0 (0%) | 0 (0%) | 0 (0%) | 0 (0%) | 0 (0%) | 0 (0%) |
| 2 | 0 (0%) | 2 (20%) | 0 (0%) | 2 (20%) | 3 (30%) | 3 (30%) | 0 (0%) | 2 (20%) | 0 (0%) |
| 3 | 2 (20%) | 2 (40%) | 2 (20%) | 4 (60%) | 4 (70%) | 3 (60%) | 0 (0%) | 0 (20%) | 2 (20%) |
| 4 | 1 (30%) | 2 (60%) | 2 (40%) | 2 (80%) | 0 (70%) | 2 (80%) | 1 (10%) | 1 (30%) | 1 (30%) |
| 5 | 1 (40%) | 0 (60%) | 1 (50%) | 1 (90%) | 0 (70%) | 0 (80%) | 0 (10%) | 0 (30%) | 0 (30%) |
| 6 | 0 (40%) | 0 (60%) | 0 (50%) | 0 (90%) | 0 (70%) | 0 (80%) | 0 (10%) | 0 (30%) | 0 (30%) |
| 7 | 0 (40%) | 0 (60%) | 1 (60%) | 0 (90%) | 0 (70%) | 0 (80%) | 0 (10%) | 0 (30%) | 0 (30%) |
