# Supplementary figures and images for: First Characterization of a Hafnia Phage Reveals Extraordinarily Large Burst Size and Unusual Plaque Polymorphism
Source: Front Microbiol. 2022 Feb 8;12:754331. doi: 10.3389/fmicb.2021.754331 (PMC8861465; doi:10.3389/fmicb.2021.754331)

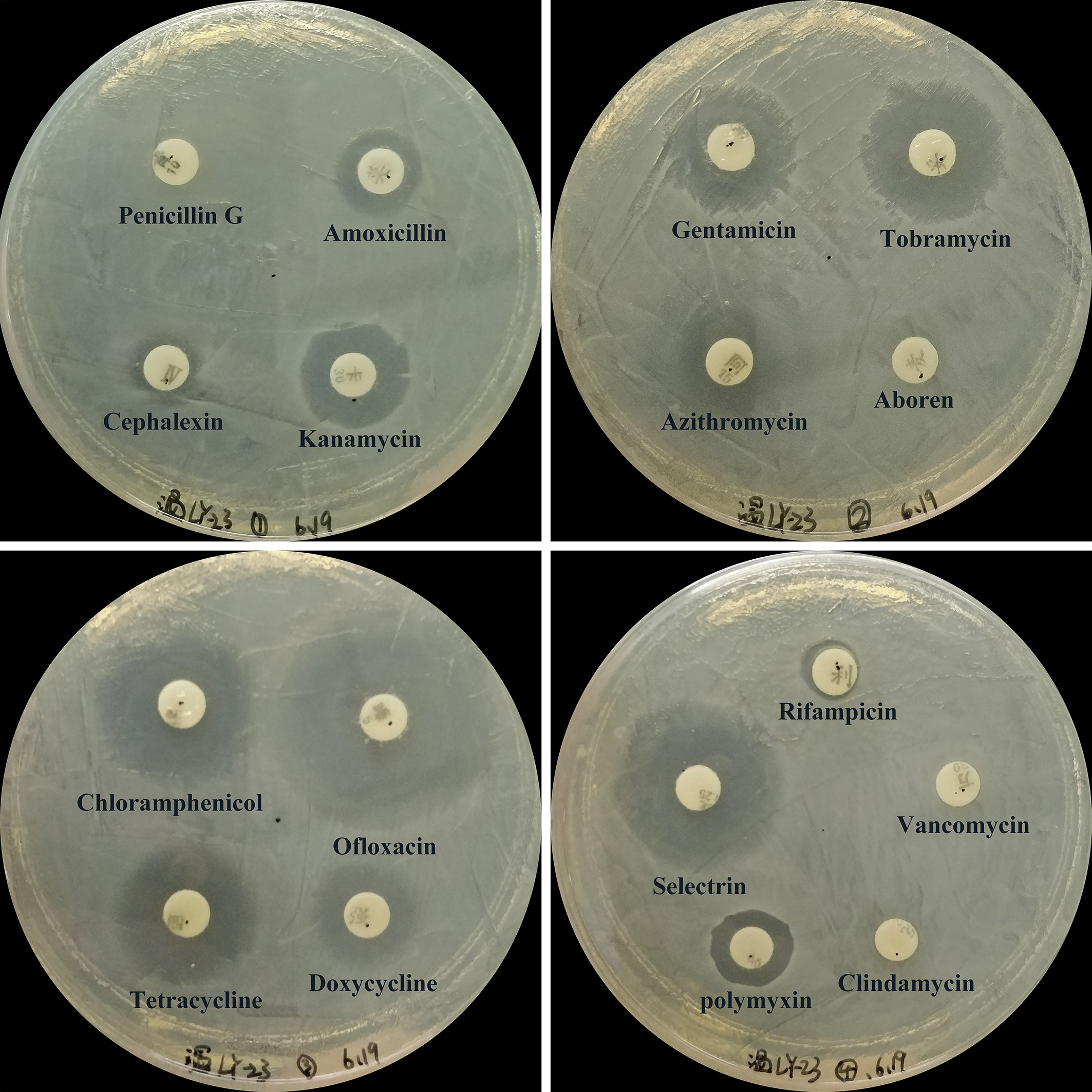

Supplement: Supplementary file 2 [file Image_1.JPEG]

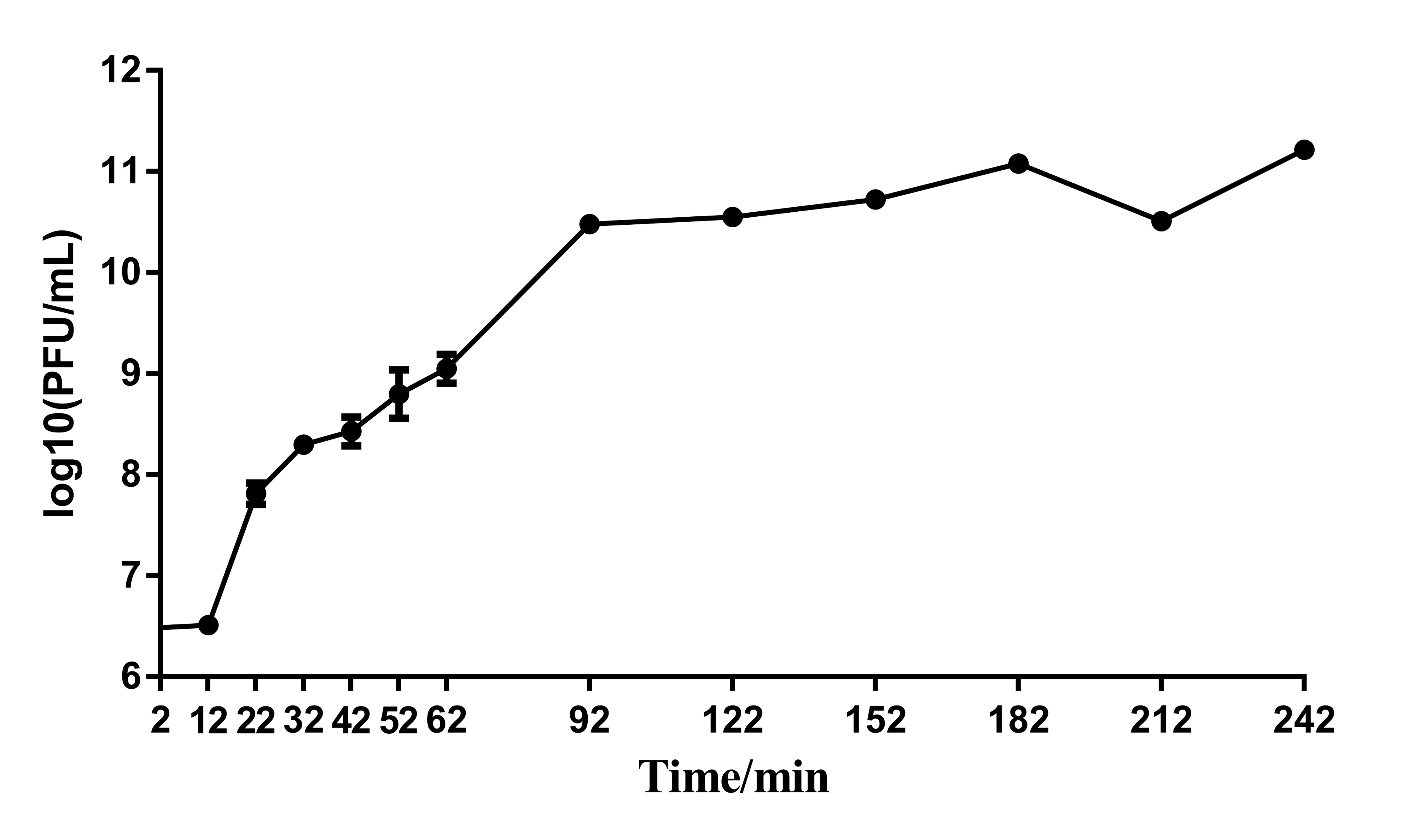

Supplement: Supplementary file 3 [file Image_2.JPEG]

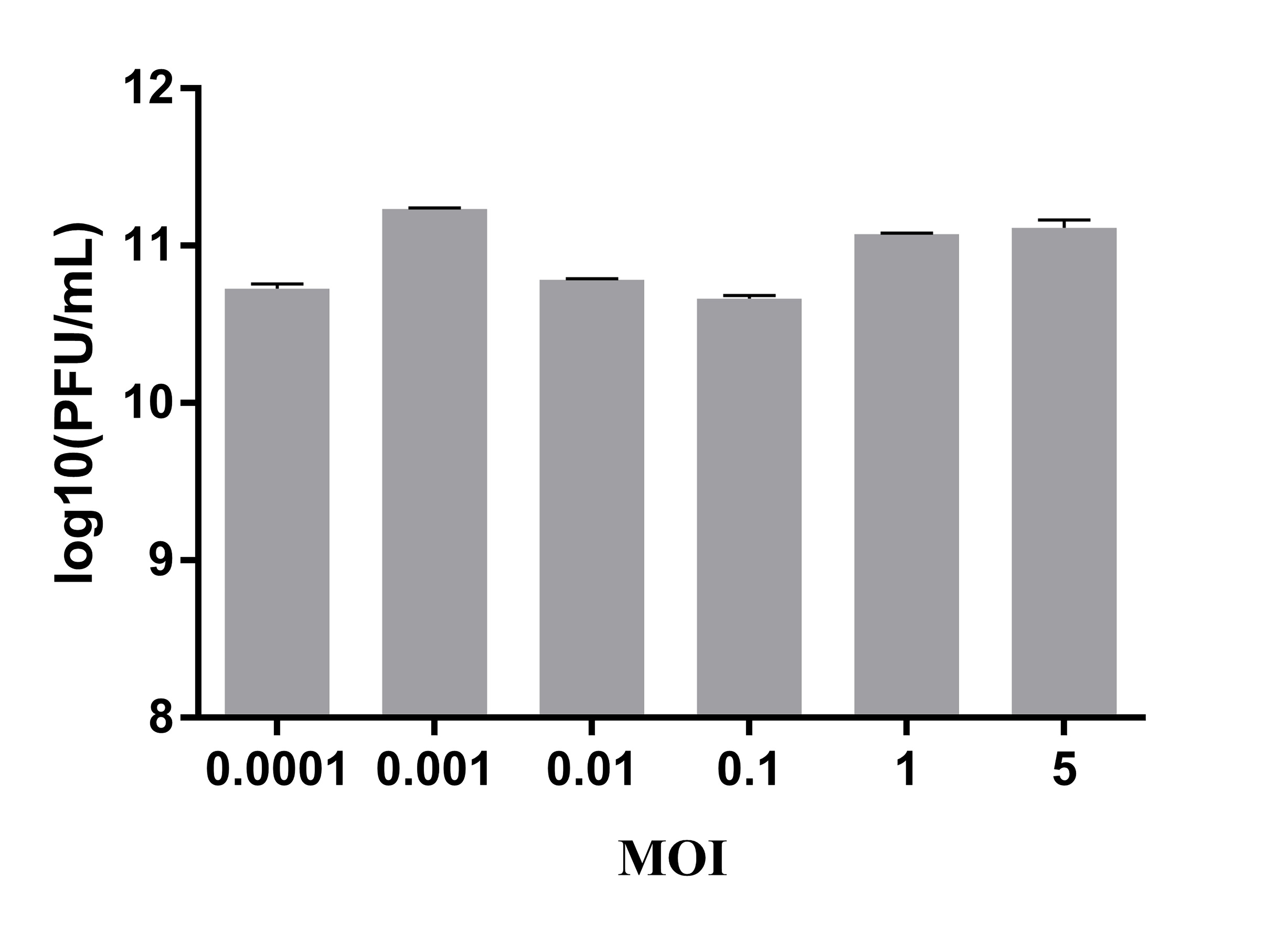

Supplement: Supplementary file 4 [file Image_3.JPEG]

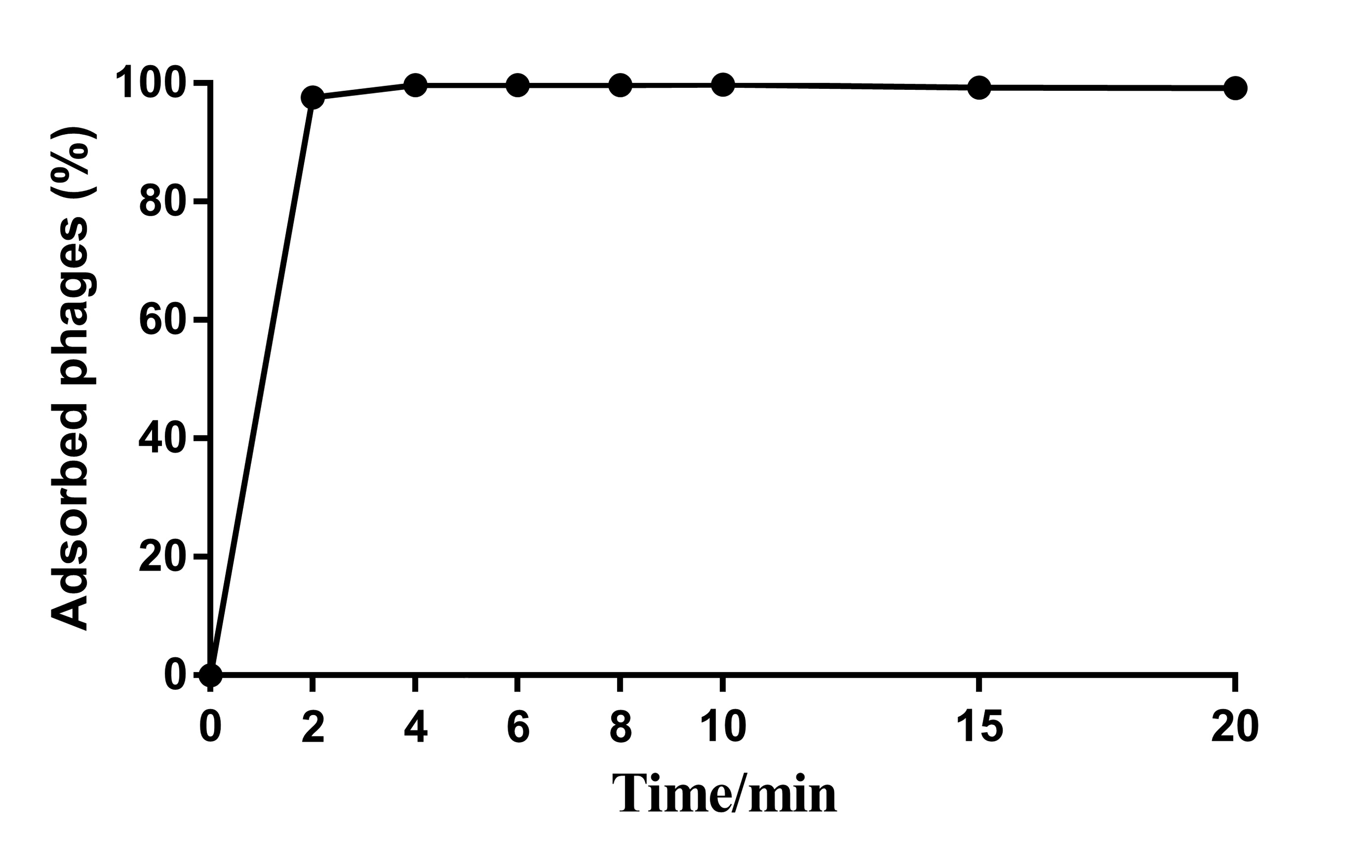

Supplement: Supplementary file 5 [file Image_4.JPEG]

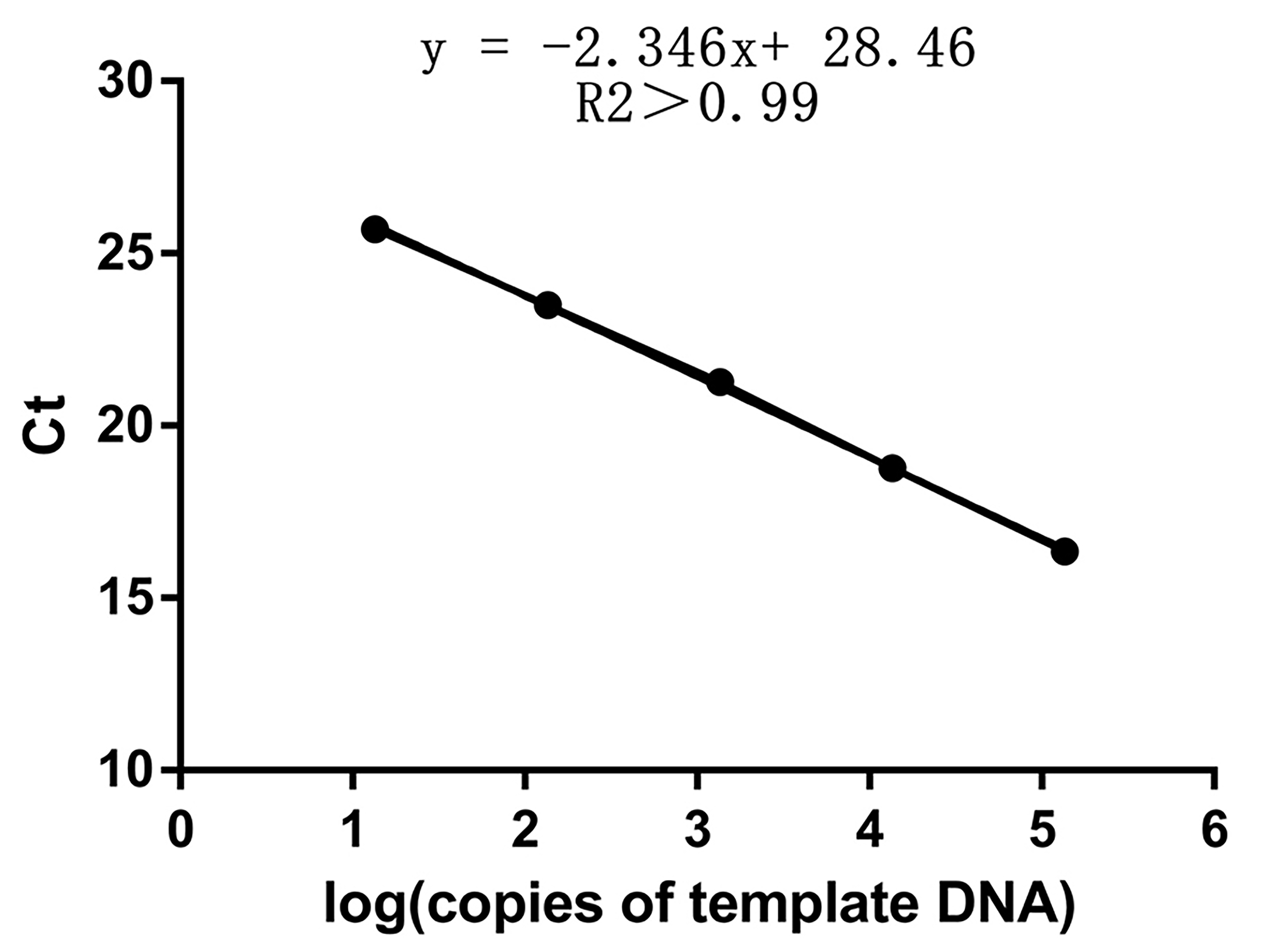

Supplement: Supplementary file 6 [file Image_5.JPEG]

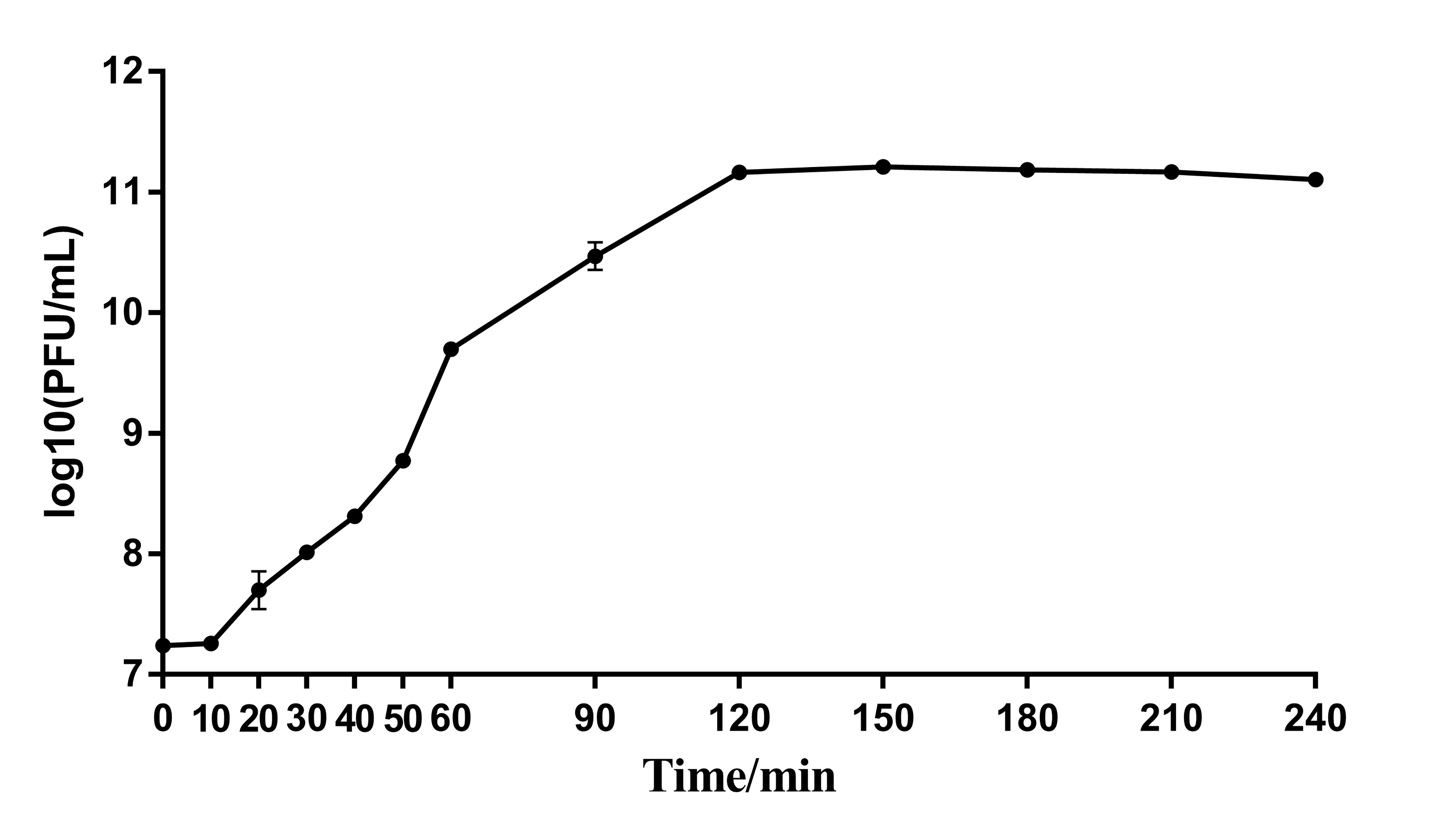

Supplement: Supplementary file 7 [file Image_6.JPEG]

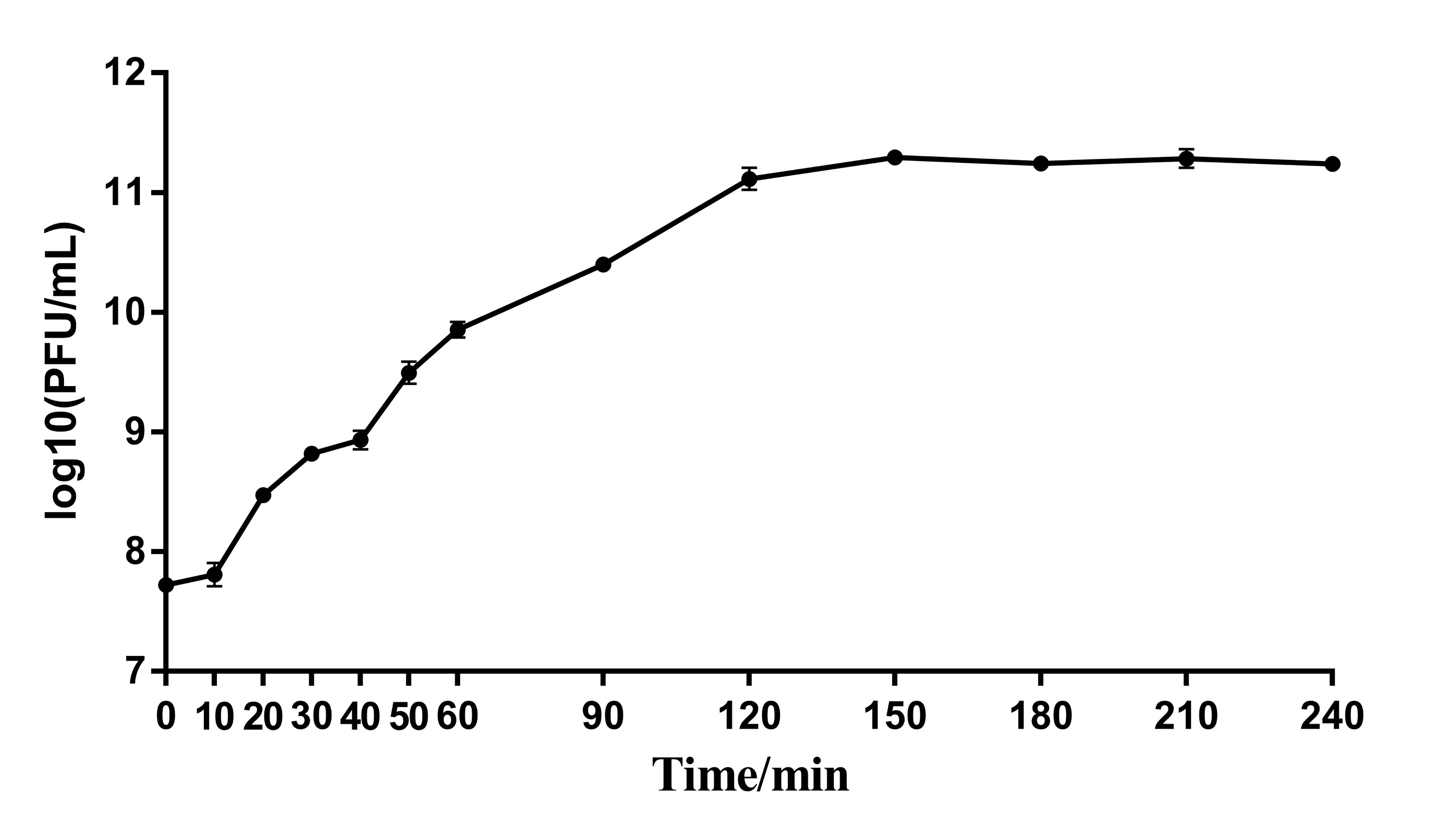

Supplement: Supplementary file 8 [file Image_7.JPEG]

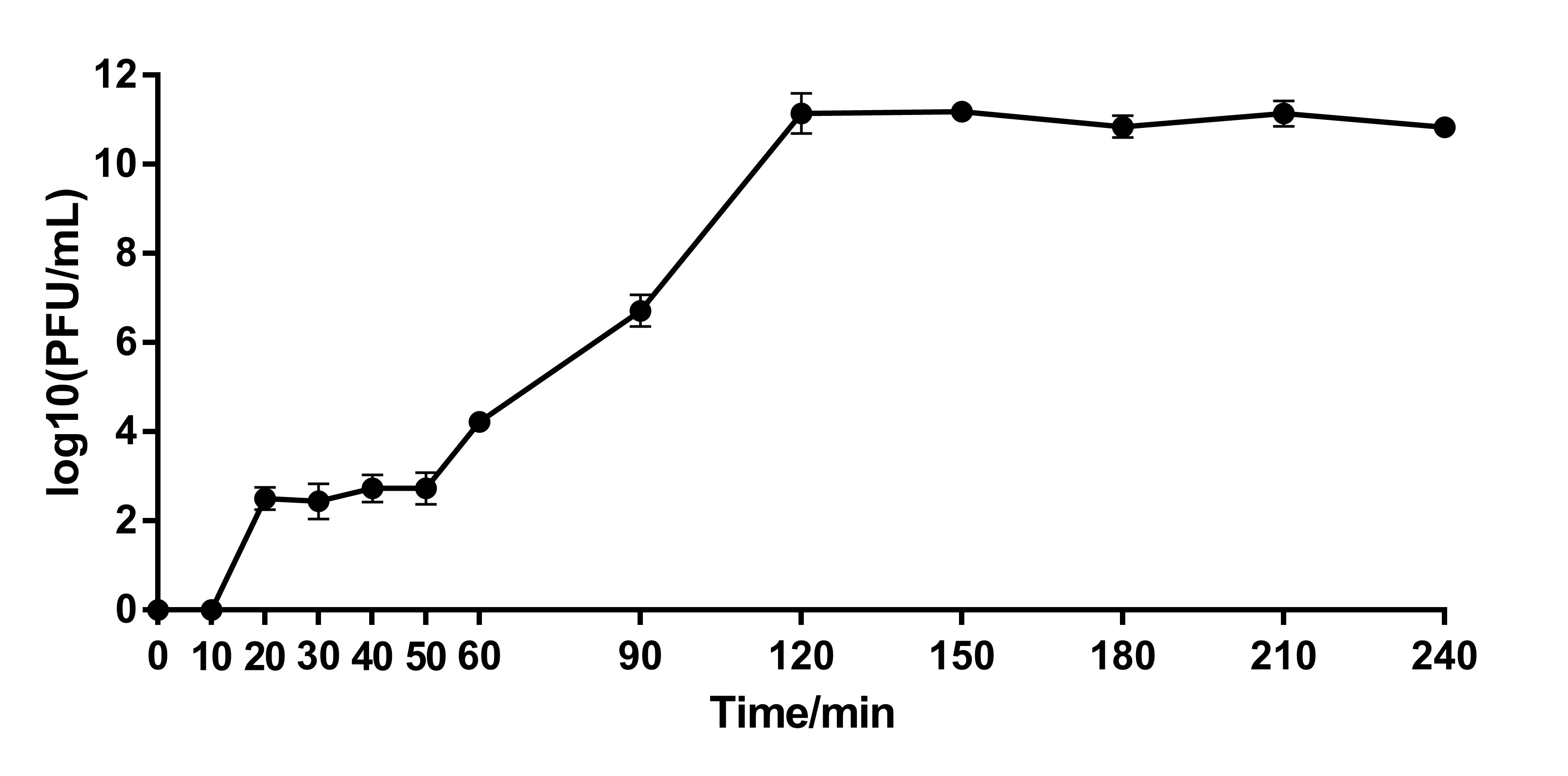

Supplement: Supplementary file 9 [file Image_8.JPEG]

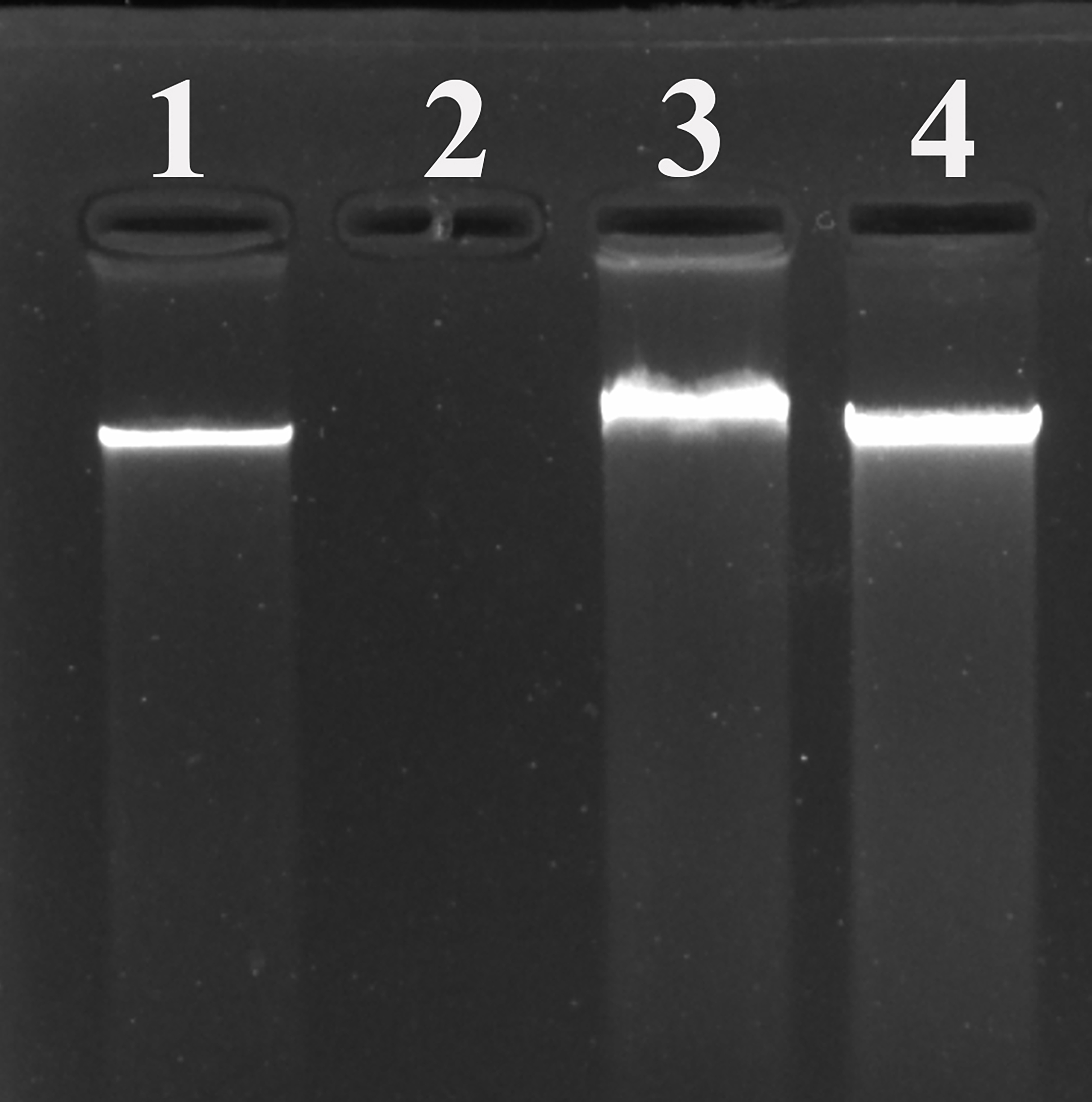

Supplement: Supplementary file 10 [file Image_9.JPEG]
